# An aging and p53 related marker: *HOXA5* promoter methylation negatively correlates with mRNA and protein expression in old age

**DOI:** 10.18632/aging.202621

**Published:** 2021-02-05

**Authors:** Laura-Kim Feiner, Sascha Tierling, Sebastian Holländer, Matthias Glanemann, Claudia Rubie

**Affiliations:** 1 Department of General-, Visceral-, Vascular- and Pediatric Surgery, University of Saarland Medical Center, Homburg 66421, Saar, Germany; 2 Department of Genetics and Epigenetics, Saarland University, Saarbrücken 66123, Germany

**Keywords:** ageing, DNA methylation, HOXA5, p53, gene expression

## Abstract

The process of aging has been associated with differential patterns of DNA methylation which relate to changes in gene expression. Hence, we aimed to identify genes with significant age-related methylation differences and to study their mRNA and protein expression profile. Genome-wide DNA methylation analysis was performed with the Illumina Infinium Methylation EPIC BeadChip Microarray on bisulfite-converted DNA prepared from monocytes derived from young (average age: 23.8 yrs) and old (average age: 81.5 yrs) volunteers that are separated by at least 50 years of age difference, n=4, respectively. Differentially methylated CpG sites were assigned to the associated genes and validated by deep sequencing analysis (n=20). Demonstrating an age-associated significant increase of methylation in the promoter region (p=1x10^-8^), Homeobox A5 (HOXA5), also known to activate p53, emerged as an interesting candidate for further expression analyses by Realtime PCR, ELISA and Western Blot Analysis (n=30, respectively). Consistent with its hypermethylation we observed significant *HOXA5* mRNA downregulation (p=0.0053) correlating with significant *p53* downregulation (p=0.0431) in the old cohort. Moreover, we observed a significant change in HOXA5 protein expression (p=3x10^-5^) negatively correlating with age and promoter methylation thus qualifying HOXA5 for an eligible p53-related aging marker.

## INTRODUCTION

The aging process is a complex physiological process leading to substantial alterations of biological functions. At the same time, aging is accompanied by a general promotion of disease risk and increased susceptibility to age-related diseases (ARDs), ultimately followed by death [1].

With increasing age a distinct remodeling of genomic DNA methylation (DNAm) patterns takes place which is characterized by a decrease in global DNAm that affects mainly CpG poor regions in the neighborhood of CpG islands (CpG shores/shelves) and also repetitive regions [2, 3]. At the same time, hypermethylation in specific regulatory regions increases, primarily at CpG islands (CGIs) within gene promoters and near gene-rich regions [4, 2]. Where this process appears to be directional, it may reflect a programmed change of the methylation code contributing to the aging process. In this context, DNA hypermethylation is known to be important for the regulation of various genes involved in crucial physiological processes like cell cycle and apoptosis, but occurs also during development, aging and tumorigenesis [5, 6].

CpGs in such genes and their respective age-related DNAm changes are used as biomarkers forming the basis of ‘epigenetic clocks’ that enable accurate biological and chronological age estimation in humans and other mammals [7–9]. While the ‘Hannum epigenetic clock’ [8] accurately estimates age based on blood methylation levels, ‘Horvath's epigenetic clock’ [7] was developed as a multi-tissue epigenetic clock based on changes at 353 age-associated CpG sites. While this clock has been applied to many mammals and human cohorts, including patients with diseases such as HIV, cancer and progeria [10–13], it was also applied to predict accelerated or delayed aging caused by lifestyle [14]. However, biological aging of blood can also be tracked by DNAm changes at just three CpG sites [15]. Intriguingly, aging-associated DNAm changes can be counteracted in induced pluripotent stem cells (iPSCs) as DNAm of iPS was shown to be significantly younger than that of corresponding primary cells. [7, 15]. While Yamanaka transcription factors (OSKM) can reverse aging-associated DNAm changes, they were recently applied to progeroid mice in short term cyclic exposure thereby restoring levels of H3K9me3 and H4K20me and increasing maximal lifespan by approximately 15% [16]. Yet, common regimens known to prolong life span like sirtuin activation, calorie restriction, CoA depletion and polyamines, seem to function through epigenetic/DNAm alterations, that affect histone acetylation, ultimately stimulating autophagy [17]. Concerning heredity transmission of DNAm, it is commonly assumed that most marks are erased and not intergenerationally inherited. However, individual epigenomic loci may not be erased or they may be restored by yet unknown mechanisms in subsequent generations [18] explaining why PBMCs of Italian semi-supercentenarians (age 105-109 years) and their off-spring showed decreased epigenetic age compared to age-matched controls [19].

In spite of all the exciting examples of progress in aging-related DNAm research and although pathological effects of altered DNAm have been connected with increasing age [20], it is still a matter of debate if and how DNAm changes contribute to aging. Then again, genome-wide DNA methylation analyses have discovered similar methylation patterns shared between the aging process and tumorigenesis and associated alterations in methylation with an increased risk for the development of tumors in the elderly suggesting that malignant transformation may even be promoted by senescence [21, 22]. In this context, it has been suggested that most methylation changes are generated in a programmed manner predisposing cells for tumorigenesis [23]. Thus, accumulation of age-associated regional hypermethylation in promoter-associated CGIs of tumor suppressor genes and the subsequent changes in gene expression may add to the phenotypic onset of many tumors [24, 21].

To date, genome-wide epigenomic profiling enables large-scale epigenetic biomarker screening for disease diagnosis and prognosis on patient-derived samples [25]. Hence, the detection of promoter hypermethylation and transcriptional silencing based on genome-wide DNA methylation analyses has significantly facilitated the detection of disease-associated candidate genes. Whole-genome arrays in combination with high-density expression array analyses have identified a number of genes that are frequently methylated and silenced in various tumor entities [26, 27]. Based on these methods, we identified various genes of which Homeobox A5 (HOXA5), an anti-angiogenic member of the HOX family, turned out to be the most interesting candidate displaying a significant age-dependent increase of DNAm correlating with a significant decrease of its gene and protein expression. Deregulation of *HOXA5* gene expression is involved in tumor predisposition and development where it may be modulated by epigenetic mechanisms [28–31]. HOXA5 is also known to increase vascular stability by the downregulation of pro-angiogenic factors and the upregulation of anti-angiogenic factors, such as tumor suppressor protein p53 [32, 33]. In addition to its well-established role in tumorigenesis, p53 has also been associated with aging and may contribute as a key factor to the protection from diseases and cancer in centenarians [34]. p53 in turn is involved in a complex network of interactions with Phosphatase and Tensin Homolog deleted on Chromosome 10 (PTEN), also an effective suppressor of cancer and a contributor to longevity, which in reciprocal cooperation promotes stability and transcriptional activity of p53, while p53 was reported to enhance *PTEN* transcription [35, 36].

Based on the knowledge that epigenetic marks, especially methylation, change with age we aimed to identify genes with significant age-related methylation differences which led to the detection of HOXA5 as a potential cancer-related candidate marker for old age.

## RESULTS

### Genome-wide DNA methylation analysis

To generate genome-scale DNA methylation profiles the Illumina Infinium MethylationEPIC BeadChip Microarray was applied which covers promoters and putative regulatory domains of all designable RefSeq genes [37]. The microarray results are based on monocytes isolated from a cohort of young (average age: 23.8 yrs, range: 22-25 yrs) and old (average age: 81.5 yrs, range: 78-89 yrs) adults separated by at least 50 years of age difference, n = 4, respectively. As the association between DNA methylation and an exposure of interest could be confounded by cellular heterogeneity [38], we have chosen the monocyte fraction of peripheral blood as the most homogeneous cell type in the blood best suited for addressing epigenetic issues.

**Figure 1:**
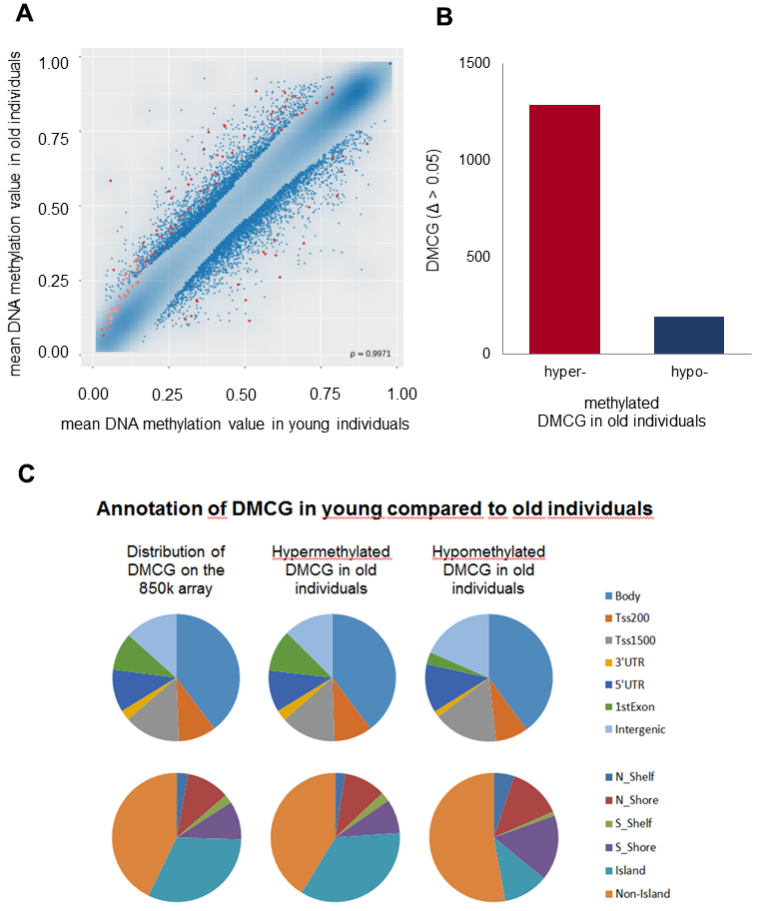
**Genome-wide DNAm analysis.** Genome-scale DNAm profiles derived from the RnBeads software package. (**A**) Scatter plot based on the mean DNAm values of each analysed CpG site of the individual trial participants compared between the young and old cohort (red dots correspond to dmCGs with p-values < 0.05). The correlation coefficient is ρ = 0.9971. (**B**) Numbers of differentially hypo- and hypermethylated CpGs (DMCG) in old compared to young individuals demonstrate distinctly more hyper- than hypomethylated DMCG in old compared to young individuals. (**C**) Hypo- and hypermethylated dmCGs are annotated in terms of gene regulatory regions (intergenic gene regions, 1st exon, 3′; and 5′; untranslated region (UTR), gene body, promoter areas: transcription start sites (TSS) 1500 and 200; upper pie charts), and CGIs (CGIs and flanking regions before (N shelf, N shore) and after (S shelf, S shore) CGIs; lower pie charts) in comparison to the overall distribution of markers on the whole 850 K array (left pie charts), respectively.

To detect differentially methylated CpG sites, also referred to as methylated quantitative trait loci (methQTLs) between the two cohorts, the microarray raw data were fed into the RnBeads software package [39]. Based on defined criteria, which comprise a measured bead count of at least 5 and a false discovery rate (FDR) adjusted p-value less than 0.05, the RnBeads software revealed 1481 differentially methylated CpGs (dmCGs) that were aligned against the hg19 annotation of the ENCODE database [40] and assigned to the associated genes and respective gene regions in which the CpG sites are located. Global methylation differences (MDs) between the trial participants were subjected to a hierarchic cluster analysis. Based on the methylation values derived from the RnBeads software package a scatter plot was created comparing the mean methylation values of each analysed CpG site of the individual trial participants between the young and old cohort as presented in Figure 1A. Although a strong correlation between the methylation values of the two participant groups was measured (ρ = 0.9971), we also found 1481 red dotted CpG-sites which fulfilled the filter criteria mentioned above with a significant MD > 5% between young and old individuals. Comparing the number of differentially hypo- and hypermethylated CpGs in the old versus the young cohort we observed that 1286 of the CpGs (86.8%) were hypermethylated whereas 195 CpGs (13.2%) were hypomethylated in the elderly (Figure 1B). Annotation of hypo- and hypermethylated CpGs in terms of gene regulatory regions revealed that a major part of dmCGs was located to the gene body while 13.6% of CpGs were located around transcription start sites as shown in the top panel of Figure 1C. In CGIs of the old cohort we found more hypermethylated CpGs (27.7%) compared to the young cohort (18.3%) as presented in the bottom panel of Figure 1C. In the old individuals we also found more hypomethylated CpGs located to non-CGI DNA sequences (Figure 1C bottom panel). With respect to their location we found 589 (38.5%) dmCGs located in the gene body, 141 (9.2%) dmCGs were located between 1 and 200 bp upstream of the transcription start sites (TSSs) and 212 (13.9%) dmCGs were located between 1 and 1500 bp upstream of the TSSs, 39 (2.5%) dmCGs were located in the 3’UTR and 160 (10.5%) dmCGs were located in the 5’UTR. 142 (9.3%) dmCGs were located in the first exon, while we found 247 (16.1%) dmCGs not associated with any transcription unit.

### Three target genes with significant aging-related MD

**Figure 2:**
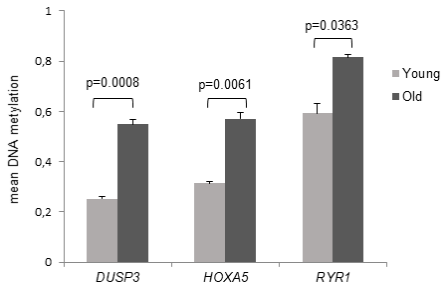
**DNAm of the target CpG sites of DUSP3, HOXA5 and RYR1.** Mean DNA methylation values of the differentially methylated CpG sites are demonstrated in young and old subjects. The associated genes were assigned to the target CpG site, respectively. Detection of mean DNA methylation was performed by microarray on bisulfite-converted DNA prepared from monocytes. Error bars denote SEM (n = 4, average age: 23.8 yrs and 81.4 yrs, respectively). P values are FDR adjusted.

Among the top ranking dmCGs we identified three CpGs with more than 20% MD associated with three genes, respectively dual specificity protein phosphatase 3 (*Dusp3*) with a MD of 29.8% (fdr-adj.p = 0.0008), *HOXA5* with a MD of 25.7% (fdr-adj.p = 0.0061) and ryanodine receptor 1 (*RYR1*) with a MD of 22.4% (fdr-adj.p = 0.0363) as presented in Figure 2. All three dmCGs indicated significant hypermethylation associated with old age.

The *HOXA5* target CpG was located 1450 bp upstream of the transcription start site (TSS) thus being associated with the promoter region. In addition, the *HOXA5* target CpG site was located in a multiple transcription factor binding region including the binding site for transcription factor RBL2. However, the target CpG of *DUSP3* was located in the 3’UTR while the target CpG of *RYR1* was situated in the gene body. The target CpG of *DUSP3* was situated in exon 3 in a multiple transcription factor binding region with annotated binding including inter alia the binding site for transcriptional repressor CTCF and transcription factor STAT1. Unlike *HOXA5* and *DUSP3* the target CpG of *RYR1* which was located in exon 13, indicated no annotated binding of regulatory proteins [40].

Hence, we aligned our 1481 dmCGs with Horvath’s clock [7] and also with an RnBeads pipeline consisting of Horvath CpGs and additional Age-Prediction- CpGs from diverse tissues. Eight of our dmCGs overlapped with the 353 CpGs of Horvath’s clock while 29 of our dmCGs overlapped with the 761 CpGs of our RnBeads pipeline. None of the three CpGs investigated here, associated with HOXA5, DUSP3 and RYR1, respectively, overlapped with either of these two data sets. A detailed table showing the position of all the 1481 differentially methylated CpG sites with indication of the assigned gene, the chromosomal location and the respective gene region as well as the overlapping dmCGs described above is provided in Supplementary Table 1.

As *HOXA5, DUSP3* as well as *RYR1* displayed significant aging-associated MDs and functional methylation has been demonstrated not only for promoter regions but e.g. also for 3‘UTRs [41], we chose to investigate all three dmCGs in more detail using local deep bisulfite sequencing analysis.

### Bi-PROF revealed significant aging-associated hypermethylation of HOXA5

**Figure 3:**
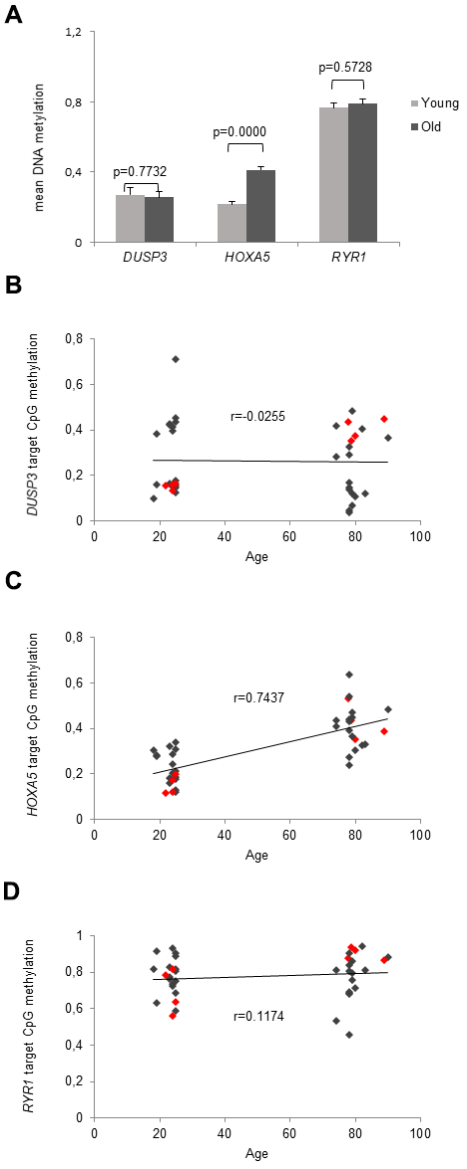
**Deep analysis of CpG sites.** (**A**) Validated methylation values of the microarray-detected CpG sites are demonstrated in young and old subjects. Detection of mean DNA methylation was performed by local deep sequencing on bisulfite-converted DNA prepared from monocytes. The associated genes are assigned to the target CpG site, respectively. Error bars denote SEM (n = 20, average age: 23.4 and 79.6 yrs, respectively). (**B**–**D**) shows the correlation between the methylation of the target CpG site of *DUSP3*, *HOXA5* and *RYR1* and the participant's age. Methylation values of the 8 individuals that were originally applied in the MethylationEPIC BeadChip Microarray are accentuated by red dots. (**B**) Depicts the correlation between the methylation of the target CpG site of *DUSP3* and the participants age showing a very low correlation coefficient (r = -0.0255). (**C**) Delineates a linear correlation between *HOXA5* methylation in the target CpG site of the *HOXA5* promoter region and the participants age showing increasing methylation levels with increasing age as reflected in the respective correlation coefficient (r = 0.7437). (**D**) Depicts the correlation between the methylation of the target CpG site of *RYR1* and and the participants age showing a very low correlation coefficient (r = 0.1174).

Based on our filter criteria used for the Bead array analysis, target CpGs of *HOXA5*, *DUSP3* and *RYR1* were selected for bisulfite profiling (Bi-PROF) [42] in a cohort of young (average age: 23.4 yrs, range: 18-25 yrs) and old (average age: 79.6 yrs, range: 75-90 yrs) adults, n = 20, respectively. Deep sequencing revealed no significant age-associated MD for *DUSP3* and *RYR1* neither for the target CpG site (Figure 3A) nor for the mean amplicon methylation.

However, examining the cohort applied in the MethylationEPIC BeadChip Microarray which consisted of 4 young and 4 old individuals (red dots Figure 3B–3D), we saw a significant aging-related MD for the dmCGs associated with *DUSP3* (red dots Figure 3B) and *RYR1* (red dots Figure 3D) also in the Bi-PROF experiments with p = 4.27x10^-5^ and p = 0.0188, respectively, indicating that the quality of the microarray and data evaluation was sufficient but sample number was too low.

In contrast, a significant age-associated MD was observed for the target CpG site (Figure 3A) and all other 13 CpGs present in the deep sequenced amplicon of *HOXA5* (p = 1x10^-8^) in the larger Bi-PROF cohort. Moreover, a linear correlation between *HOXA5* methylation in the target CpG site of the promoter region and the participants’ age was observed as depicted in Figure 3C. Increasing methylation correlated with increasing age as reflected in the respective correlation coefficient (r = 0.7437).

### Significant aging-associated down-regulation of *HOXA5* mRNA expression

**Figure 4:**
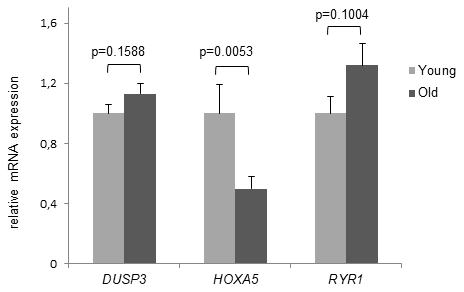
**Age- dependent HOXA5 mRNA decline**. mRNA expression levels of *DUSP3*, *HOXA5* and *RYR1* are demonstrated in young and old subjects. While there was no significant difference in mRNA expression demonstrated for *DUSP3* and *RYR1*, *HOXA5* mRNA levels are significantly down-regulated in monocytes of old blood donors. Detection of mRNA expression was performed by qRT-PCR on total cellular RNA prepared from monocytes. Error bars denote SEM (n = 30, average age: 23.0 yrs and 81.1 yrs, respectively).

Although deep sequencing revealed no significant age-associated MD for *DUSP3* and *RYR1*, in the next set of experiments we compared *HOXA5, DUSP3* and *RYR1* DNAm results to the gene expression level. mRNA expression was studied in the same cohorts of young and old volunteers that were included in Bi-PROF as well as in 10 additional volunteers, respectively (average age: 23.0 yrs, range: 18-25 yrs, n = 30 and average age: 81.1 yrs, range: 75-90 yrs, n = 30). As presented in Figure 4, *HOXA5* mRNA expression is significantly downregulated in monocytes from old subjects compared to the young cohort (p = 0.0053) while *DUSP3* and *RYR1* mRNA expression shows no significant difference as was expected from the deep sequencing results. Since DNAm of *HOXA5* in the promoter region is significantly increased in old subjects, significant down-regulation of *HOXA5* mRNA expression corresponds well with these results. Therefore, only *HOXA5* was chosen for further investigation of gene expression on the protein level.

### Significant aging-associated down-regulation of HOXA5 protein expression

**Figure 5:**
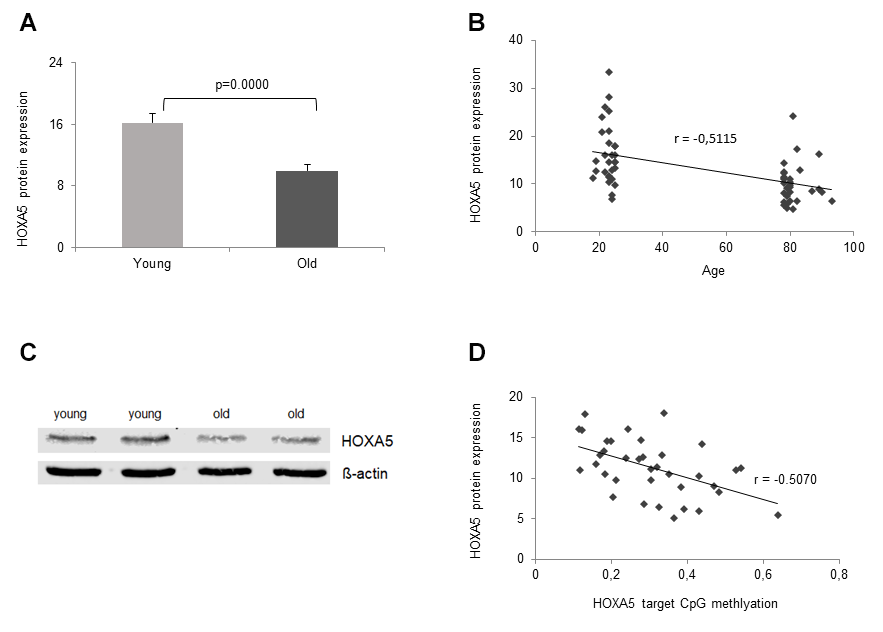
**Age- dependent HOXA5 protein decline.** HOXA5 protein levels are significantly down-regulated in monocytes from old blood donors. (**A**) HOXA5 protein quantities were determined by ELISA in total cell lysates of monocytes isolated from young and old blood donors expressed as ng per mg total protein. Error bars denote SEM (n = 30, average age: 23.0 yrs and 81.1 yrs, respectively). (**B**) Delineates a linear correlation between HOXA5 protein expression and the participants age showing decreasing protein levels with increasing age as reflected in the respective correlation coefficient (r = -0.5357). (**C**) Twenty-five micrograms of total cell lysates from monocytes isolated from young and old blood donors were subjected to immuno-protein gel blotting with rabbit monoclonal anti-human HOXA5 antibody ab140636 (1:500), recognizing the HOXA5 protein at 42 kDa (predicted molecular weight 29 kDa). ß-actin served as loading control. (**D**) delineates a linear correlation between HOXA5 expression (ng per mg total protein) and *HOXA5* methylation in the target CpG site of the promoter region showing decreasing protein levels with increasing methylation as reflected in the respective correlation coefficient (r = -0.5070).

Next, we were intrigued to find out, if ageing-related significant down-regulation of *HOXA5* mRNA expression is translated into protein expression. Therefore, HOXA5 protein expression was studied in the same cohorts of young and old volunteers that were included in the mRNA expression analysis, n = 30, respectively. In accordance with significant down-regulation of *HOXA5* mRNA expression in the old cohort, HOXA5 protein levels in the elderly were also significantly reduced (p = 3x10^-5^, Figure 5A). Further, a linear correlation between HOXA5 protein expression and the participants age was observed as depicted in Figure 5B. Decreasing protein levels were shown to correlate with increasing age as reflected in the respective correlation coefficient (r = -0.5357). As assessed by western blot analysis, HOXA5 protein levels also correlate negatively with increasing age (Figure 5C). As summarized in Figure 5D HOXA5 protein expression is also negatively correlated with promoter methylation which is reflected in the respective correlation coefficient (r = -0.5070). Altogether, our findings suggest that age-related up-regulation of *HOXA5* methylation may lead to the down-regulation of *HOXA5* mRNA and protein expression in old age.

### Significant aging-associated correlation of *HOXA5* expression with *p53* and *PTEN*

**Figure 6:**
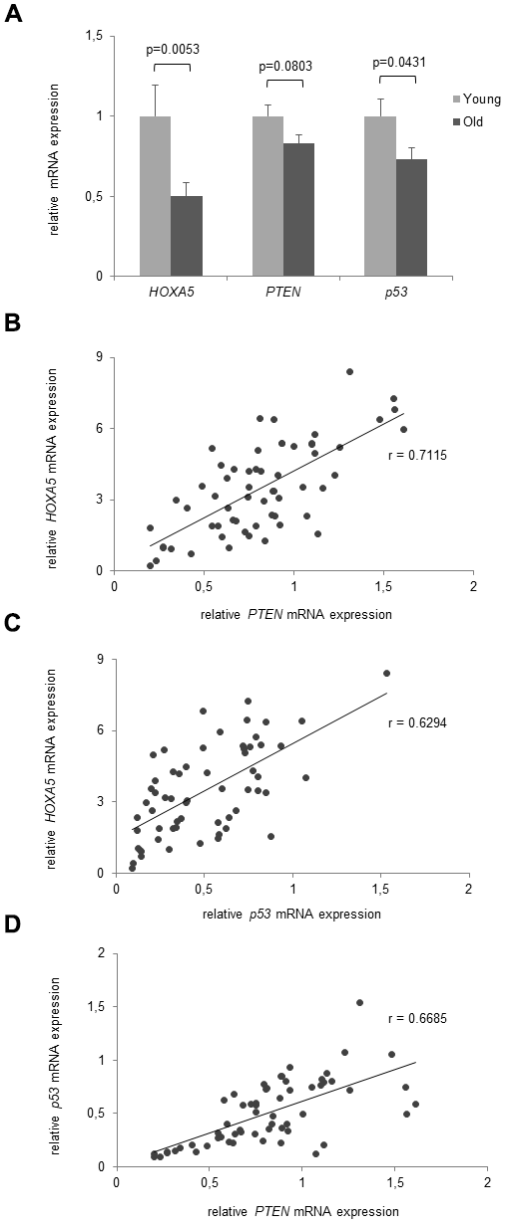
**Age- dependent correlation of HOXA5, PTEN and p53 mRNA expression.** (**A**) mRNA expression levels of *HOXA5*, *PTEN* and *p53* are downregulated in the old subjects compared to the young cohort. (**B**) Moderate to strong linear correlations were observed between *HOXA5* and *PTEN* mRNA expression (r = 0.7115) (**C**) between *HOXA5* and *p53* mRNA expression (r = 0.6294), and (**D**) between *p53* with PTEN mRNA expression (r = 0.6685). Detection of mRNA expression was performed by qRT-PCR on total cellular RNA prepared from monocytes. Error bars denote SEM (n = 30, average age: 23.0 yrs and 81.1 yrs, respectively).

As Hoxa5 is known to interact with p53 and PTEN we investigated their age-dependent correlation on the gene and protein expression level. Expression was studied in the same cohorts of young and old volunteers that were included in the previous expression experiments (average age: 23.0 yrs, range: 18-25 yrs, n = 30 and average age: 81.1 yrs, range: 75-90 yrs, n = 30). As presented in Figure 6A, *HOXA5*, *p53* and *PTEN* mRNA expressions are downregulated in monocytes from the old subjects compared to the young cohort. However, while *HOXA5* and *p53* were shown to be significantly down-regulated (p = 0.0053 and p = 0.0431, respectively), *PTEN* mRNA expression only showed a tendency of down-regulation without significance (p = 0.0803). Further, moderate to strong linear correlations were observed between *HOXA5* and *p53* mRNA expressions (r = 0.6294) and between *HOXA5* and *PTEN* (r = 0.7115) mRNA expressions as depicted in Figure 6B, 6C, respectively. Comparing *p53* with *PTEN* mRNA expressions, we also observed a moderate to strong linear correlation (r = 0.6685) as shown in Figure 6D.

**Figure 7:**
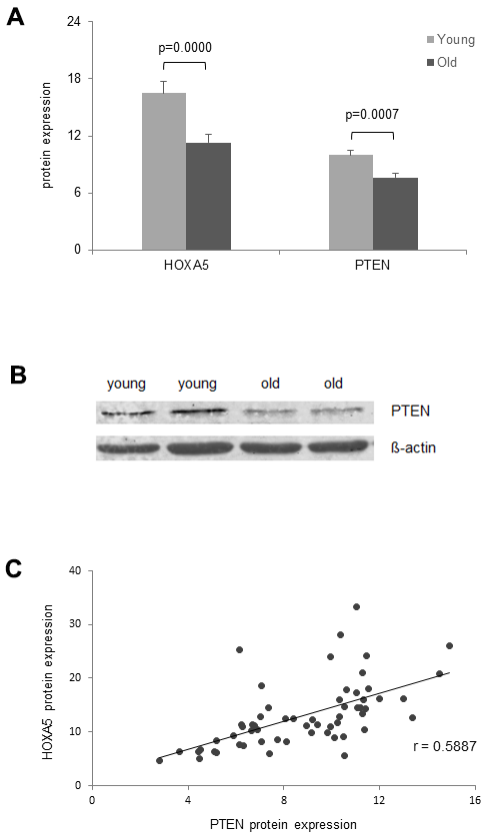
**Age- dependent correlation of HOXA5 and PTEN protein expression.** (**A**) HOXA5 and PTEN protein quantities are significantly down-regulated in the old cohort as determined by ELISA in total cell lysates of monocytes isolated from young and old blood donors expressed as ng per mg total protein. Error bars denote SEM (n = 30, average age: 23.0 yrs and 81.1 yrs, respectively). (**B**) Fifteen micrograms of total cell lysates from monocytes isolated from young and old blood donors were subjected to immuno-protein gel blotting with primary rabbit monoclonal anti-human PTEN antibody (1:10000), recognizing the 47 kDA PTEN protein. ß-actin served as loading control. (**C**) Delineates a moderate linear correlation between HOXA5 and PTEN protein expression as reflected in the respective correlation coefficient (r = 0.5887).

On the protein expression level, Hoxa5 was compared only to PTEN expression, since p53 is not expressed in monocytes as described in the GeneCards Human Integrated Protein Expression Database (HIPED) [43]. In line with HOXA5 protein expression, we observed significant down-regulation of PTEN protein expression in the old cohort as assessed by ELISA (Figure 7A) and western blot analysis (Figure 7B). Correlation analysis between HOXA5 and PTEN protein expression revealed a moderate linear correlation as reflected in a correlation coefficient of r = 0.5887 (Figure 7C).

## DISCUSSION

In the course of aging epigenetic mechanisms play an important role and in particular, changes in DNA methylation are associated with aging [44]. Here, we were interested to identify specific genomic loci differentially methylated in the aging process which may basically be assessed by genome-wide analysis and single gene analysis. Hence, it was the aim of this study to identify specific genes or regulatory regions with significant age-related MDs and to study whether altered DNAm may be accompanied by altered mRNA or protein expression.

Three dmCGs with MD > 20% were identified which were associated with *HOXA5*, further *RYR1*, known to code for a ryanodine receptor in skeletal muscles [45] and *DUSP3*, which is implicated in cancer and negatively regulates members of the mitogen-activated protein (MAP) kinase superfamily [46]. As all three genes displayed a significant increase of DNAm with age in the MethylationEPIC BeadChip Microarray, they were all considered in a subsequent Bi-PROF analysis. While the target CpG site of *HOXA5* revealed a significant age-associated MD, the microarray results of *RYR1* and *DUSP3* were not confirmed by the Bi-PROF. A significant age-associated MD for *DUSP3* and *RYR1* was neither confirmed for the target CpG sites (p = 0.7732 and 0.5728, respectively) nor for the mean amplicon methylation. However, the cost intensive genome-wide methylation analysis was based on only 4 participants of the young and 4 participants the old group. These 8 individuals showed a significant aging-related MD for *DUSP3* and *RYR1* in the Bi-PROF experiments with p = 4.27x10^-5^ and p = 0.0188, respectively, confirming the microarray results. Therefore, the discrepancy concerning MD significance for *DUSP3* and *RYR1* between the genome-wide methylation analysis and the Bi-PROF may be explained by the low number of participants that the microarray was based on. In contrast, a significant age-associated MD was observed not only for the target CpG site of *HOXA5* (p = 1x10^-8^) but also for all other 13 CpG sites which were part of the amplicon and investigated in Bi-PROF. In addition, the corresponding promoter area exhibits clustered transcription factor binding sites for POLR2A, EZH2 and RBL2 [40]. POLR2A encodes the largest subunit of RNA polymerase II, the polymerase responsible for synthesizing mRNA while EZH2 participates in H3K27 methylation and thus in transcriptional repression [47]. Also, transcription factor RBL2 binds as a transcriptional repressor to methylated DNA where it recruits chromatin-modifying enzymes to the promoter which leads to epigenetic transcriptional repression hereby reinforcing the effect of shutting down the gene. The presence of these clustered transcription factor binding sites indicates that the target CpG as well as the adjacent *HOXA5* CpG sites are located in a regulatory region in the promoter region. Moreover, in the region of the *HOXA5* target CpG site histone marks H3K27me3 and H3K27Ac are typically found also indicating that the target CpG is located within active regulatory elements. Also, Shchukina et al. recently reported (Shchukina et al., 2020, unpublished data) hypermethylation events are associated with H3K27me3 in the CpG islands near promoters of basely expressed genes which is consistent with our results as we demonstrated hypermethylation in a CpG island near a promoter region that is associated with H3K27me3 and leading to a lower expression of the associated gene *HOXA5*. Therefore, we assume that DNAm in this area has a functional influence on mRNA expression. While DNAm is a critical component of the regulatory network controlling gene expression, it may exert different influences on gene activities in different genomic regions depending on the underlying genetic sequence [48].

With regard to the 353 CpGs of the Horvath Clock and the 761 CpGs of the RnBeads pipeline, there was no match with the three CpGs examined, although the HOXA5 target CpG site showed a significant age-related difference with regard to methylation in both the MethylationEPIC BeadChip Microarray and in the validating Bi-PROF. The absent overlap may be explained by the fact that the Horvarth Clock and the RnBeads age-prediction pipeline are composed of CpG sites that have an age-associated methylation difference in all human tissues. However, the dmCGs we found refer only to human monocytes indicating that the HOXA5 target CpG site may not be methylated equally with age in all tissues explaining why we did not observe an overlap with one of the two databases. As the two other target CpGs were not validated by the Bi-PROF analysis we consequently could not observe a match with the two data sets.

Based on the assumption that differential DNAm in a regulatory promoter region has downstream consequences for gene expression and regulation, *HOXA5* was further investigated on the gene expression level where we expected a regulatory effect. Our presumption was confirmed as our results demonstrated significant down-regulation of *HOXA5* mRNA expression in the old cohort. DNAm has also been associated with down-regulation of *HOXA* genes in various cancer entities, e.g. *HOXA* genes were shown to be inactivated by hypermethylation in Myeloid and Lymphoid Malignancy [28]. Also, for HOXA5 it was demonstrated that increased DNA methylation in the HOXA5 promoter region correlates with decreased expression of the gene during tumor progression [30]. While the majority of gene promoters reside within CGIs their location is evolutionary highly conserved to regulate gene expression by regulating the chromatin structure and transcription factor binding [49]. Also, the methylation of CpG shores is highly correlated with reduced gene expression. Yet, positive associations of DNA methylation to gene expression have also been reported, i.e. in prostate cancer DNA hypermethylation in the promoter region was associated with upregulated gene expression [50] suggesting a more diverse mechanism of epigenetic regulation.

While our results demonstrate aging-associated hypermethylation and down-regulated mRNA expression of *HOXA*5, conclusions concerning a causal link between the aging-associated *HOXA*5 methylation and gene expression as well as the causal effects of *HOXA*5 methylation on regulatory activity remain at this point rather indirect. However, as summarized above there are various indicators that the area around the target CpG site of the *HOXA5* promoter is a regulatory region and therefore, it may be assumed that the aging-associated hypermethylation observed for *HOXA5* is causally related to the decrease in gene expression with increasing age. Yet, a direct proof for the causal relationship between DNA methylation and gene regulation may only be achieved by functional approaches like an episomal reporter approach [51], endogenous editing [52] or Methyl-Spec-seq [53]. However, such experiments will go beyond the scope of data presented here and are not mandatory as the presence of the regulatory region in the promoter area of *HOXA5* confers ample evidence for a causal link between *HOXA5* DNAm and mRNA expression.

Belonging to a large gene family of homeodomain-containing transcription factors HOXA5 is part of a regulatory network involved in embryonic development and cellular differentiation as well as in various crucial cell functions and human diseases like diabetes and cancer where deregulated *HOX*A5 gene expression has been described causally in tumor predisposition and in various stages of tumor progression [54, 55]. HOXA5 also blocks angiogenesis and its sustained expression was shown to promote the down regulation of many pro-angiogenic genes [33]. In addition, HOXA5 also upregulates the expression of anti-angiogenic genes like p53, one of the most frequently inactivated tumor suppressor genes in human cancers [56]. As p53 is known to interact with PTEN in a reciprocal cooperation modus to up-regulate each other [35, 36], we also investigated the age-dependent correlation of p53 and PTEN and their correlation with HOXA5 expression in our young and old cohorte. On the mRNA level we observed significant down-regulation of p53 and a tendency of down-regulation for PTEN in the old cohort as well as a moderate to strong linear correlation with HOXA5 expression for both genes indicating that all three genes are down-regulated with increasing age. Also, on the protein level we observed significant down-regulation of PTEN and a moderate linear correlation for PTEN with HOXA5 expression. We could not monitor p53 as it is not expressed in monocytes [43]. Hence, *p53* and *PTEN* correlated with *HOXA5* expression and *p53* also correlated with *PTEN* mRNA expression in our study which is well in line with studies demonstrating an increase of p53 stability, transcriptional activity and enhanced p53 protein levels due to direct binding to PTEN or indirect binding via transcriptional coactivator p300/CBP [35, 57]. Yet, up-regulation of p53 in the absence of PTEN [58] and HOXA5 regulated PTEN down-regulation in murine hemangioma cell lines has been shown [59]. However, such studies have only been performed in murine systems under diseased conditions like induced cancer and not under physiological conditions in human volunteers.

To date, HOXA5 has been implicated in p53 regulation in multiple cancer entities. Hence, HOXA5 promotes osteosarcoma cell apoptosis and the suppression of lung cancer cell invasion through the p53 pathway [60, 32]. Likewise, HOXA5 was reported as a tumour suppressor in breast cancer where it transactivates p53 transcription through binding at the p53 promoter [61]. Notably, the *HOXA*5 promoter region is methylated in most p53-negative breast tumour specimens where a compromised HOXA5 function was shown to limit p53 expression, but also in other cancer entities a correlation between DNAm and *HOXA5* expression has been described [30, 61]. Thus, HOXA5 has been discussed as a differential epigenetic biomarker between malignant and benign biliary tissues [62], while downregulation of the HOXA5 gene by aberrant promoter methylation was described in the vast majority of NSCLCs [31] and shown also for the development and progression of AML [28, 63].

Recently, HOXA5 hypermethylation has not only been associated with cancer as an aging-associated disease, but also with the age of colorectal cancer patients [55]. However, to date not many studies report on aging-related *HOXA5* DNAm or mRNA expression, especially not under physiological conditions in trials with healthy study participants. An early study described aging-related restricted expression of homeobox genes thereby distinguishing fetal from adult human smooth muscle cells [64], while a more recent study implicating HOXA5 in aging demonstrated HOXA5 to be differentially expressed in photoaging of skin [65]. When exploring a molecular relationship between aging and replicative senescence, Wagner et al., detected reduced *HOXA5* expression in mesenchymal stem cells (MSCs) [66]. Hence, expression of many homeobox transcription factors was found to be regulated upon aging and most of these were age-repressed in MSCs. Subsequently, DNAm changes in MSCs upon aging were investigated and *HOXA5* |was found to be hypermethylated in MSCs from old donors [67].

Consistent with these earlier findings, our results provide evidence that in human aging *HOXA5* DNAm significantly increases with age. Different from studies in MSCc we conducted our studies in monocytes from young and old individuals separated by a minimum of 50 years. Hence, we not only demonstrated HOXA5 hypermethylation in old age but also substantiated that it is attended by a simultaneous decline of HOXA5 mRNA expression which is translated into protein expression. Yet, it is not imperative to conclude from the methylation status of a gene to its protein expression. However, for HOXA5 we found that protein expression is negatively correlated with age and methylation thus qualifying HOXA5 for an eligible aging marker. Therefore, our findings integrating the various surveys presented above, support the view that aging forms a major risk factor for *HOXA5* DNAm which entails down-regulation of *HOXA5* mRNA and protein expression leading to the down regulation or loss of p53 expression. Lack of HOXA5 expression resulting in loss of p53 expression in turn promotes the development and progression of various cancers. Hence, we conclude that *HOXA5* hypermethylation in advanced age contributes to p53 down-regulation and in that context also to the increasing age-dependent cancer risk in the elderly.

## MATERIALS AND METHODS

### Patient selection

We analysed 60 samples obtained from peripheral blood comprising 30 samples from donors with an average age of 23.0 yrs, range: 18-25 yrs and 30 blood samples from donors of an average age of 81.1 yrs, range: 75-90 yrs. The young cohort and 11 volunteers of the old cohort presented without any known disease background while 19 volunteers of the old blood cohort presented with a minor disease background such as incisional hernia, aneurysm, hemorrhoids, varicose veins and thoracic stomach. The clinical data and patient characteristics were obtained from the clinical and pathological records prospectively. Blood samples of the young cohort were retrieved from volunteers at the University of the Saarland Medical Center, blood samples of the old cohort were collected from volunteer healthy controls that were identified during routine check-up examinations and patients at our clinic. All donors were unrelated individuals of Central European Caucasian ethnicity. Informed consent for blood sampling was obtained from all blood donors. The study was approved by the ethics committee of the medical association of the Saarland (code number 241/19).

### Monocyte isolation from whole blood samples

Peripheral Blood Mononuclear Cells (PBMC) were purified from whole blood by density gradient centrifugation using the Biocoll Solution (Cat No. 6715, Biochrom, Berlin, Germany) according to the manufacturer´s instructions. Monocytes were isolated from PBMCs by positive selection with magnetic beads using the EasySepTM Human CD14 Positive Selection Kit II (Cat No. 17858, Stemcell Technologies, Cologne, Germany) according to the manufacturer’s protocol.

### DNA, RNA and protein isolation from PBMC

For DNA extraction from monocytes the NucleoSpin® Tissue Kit (Cat No. 740952, Machery-Nagel, Düren, Germany), for RNA extraction the NucleoSpin® miRNA Kit (Cat No. 740971, Macherey-Nagel, Düren, Germany) was applied. Proteins were isolated using RIPA buffer (Cat No. 10017003, Thermo Fisher Scientific, Waltham, Massachusetts, USA), phosphatase arrest (Cat No. 786-782, G Biosciences, St. Louis, USA) and protease arrest (Cat No. 786-108, G Biosciences, St. Louis, USA).

### Bisulfite treatment

500 ng of genomic DNA were treated with sodium bisulfite using the EZ DNA Methylation-Gold Kit (Cat No. D5005, Zymo Research, Irvine, California, USA) according to the manufacturer’s protocol.

### Infinium MethylationEPIC BeadChip microarray

Bisulfite-treated DNA was subjected to Illumina Infinium MethylationEPIC BeadChip array according to the manufacturer’s protocol. This microarray measures DNA methylation levels at > 850.000 CpG sites and covers promoters and putative regulatory domains of all designable RefSeq genes (Illumina’s Infinium MethylationEPIC BeadChip Microarray, Cat No. WG-317-1001, Illumina, San Diego, California, USA). For imaging the fluorescently stained chips an Illumina HiScan scanner was applied.

### Bisulfite profiling

Local deep bisulfite sequencing follows the workflow as published by Gries et al., [42]. Briefly, amplicons containing the target CpG site were generated using region-specific primers (Supplementary Table 2, final concentration 0.167 μM each) with the Illumina universal adaptor sequences attached at their 5´-ends. Next, PCR products were purified with Agencourt AmpureBeads and pooled in an equimolar ratio. After cluster formation the PCR products were sequenced on a MiSeq instrument benchtop sequencer (Illumina) with the sequencing-by-synthesis technology [68] according to the manufacturer’s instructions aiming at 20,000 reads per amplicon.

### Data analysis

For pre-processing genome-wide methylation raw data R statistics software suites minfi and RnBeads were applied [69, 39] in order to extract the data, subtract the background, and to normalize the data with internal controls present on the chips. Only CpGs with fdr-adjusted p-value < 0.05 and a minimum MD of 5% in all samples were included (n = 1,481 of 853,307) and all samples were analyzed as individual samples (n = 1).

After Bi-PROF DNAm level and patterns were evaluated employing multiple sequence alignment based on the BiQ Analyzer HT [70]. All samples were examined as individual samples (n = 1). All reads with a fraction of unrecognized CpG sites of more than 10% were eliminated and data presented as mean ± SD. Samples identified in differential methylation analyses were assigned to corresponding gene IDs according to Illumina annotation data.

### Single-strand cDNA synthesis

For HOXA5 cDNA synthesis the High Capacity cDNA Reverse Transcription Kit (Cat No. 4368814, Thermo Fisher Scientific) was applied to reversely-transcribe 1 μg of each total RNA sample in a final reaction volume of 20 μl containing 10x TaqMan RT buffer, 25X dNTP Mix and 50 U/μL Multiscribe RT, 10x random primers, 1 μg RNA and nuclease-free water. The cycler conditions were 10 min at 25° C, 120 min at 37° C and 5 min at 85° C.

### Quantitative real-time PCR

qRT-PCR for mRNA detection was performed using 10 μl 2x Taqman Universal PCR Master Mix II and 1 μl Taqman gene expression assay (*HOXA5* - Cat No. Hs00430330_m1, *DUSP3* - Cat No. Hs01115776_m1, *RYR1* - Cat No. Hs01062613_m1, *p53* - Cat No. Hs01034249_m1 and *PTEN* - Cat No. Hs02621230_s1, Thermo Fisher Scientific), 8 μl RNase-free water and 1 μl cDNA template (50 ng/μl). Duplicates were run for all reactions together with no template controls and an additional control for DNA contamination where the reverse transcriptase was omitted. As a detection system the ABI Prism 7900 sequence detector (Thermo Fisher Scientific) was programmed to an initial step of 10 min at 95° C, followed by 40 thermal cycles of 15 s at 95° C and 10 min at 60° C. The log-linear phase of amplification was monitored to obtain CT values for each RNA sample. The expression level of the respective mRNAs was analyzed in relation to the levels of housekeeping gene POLR2A (Cat No. Hs00172187_m1, Thermo Fisher Scientific). Conversion of the individual CT values to the linear form was performed according to the 2^–∆∆CT^ method [71] and the relative standard curve method.

### Enzyme-linked immunosorbent assay (ELISA)

The expression levels of HOXA5 and PTEN proteins in monocytes were quantified by sandwich-type ELISA with Human HOXA5 ELISA Kit from My Biosource (Cat No. MBS9325409, San Diego, California, United States) and Human PTEN ELISA Kit (Cat No. ab206979, Abcam, Cambridgde, UK) according to the manufacturer's protocol. Samples were assayed in duplicate with all values calculated as the mean of 2 measurements. For measurement of protein expression levels, the absorbance at 450 nm (with 562 nm reference wavelength) was read in a 96-well microtiter plate reader. The HOXA5 protein concentration from each cell lysate was normalized to the total protein content of each sample.

### Western blot analysis

Total protein (15 μg/lane) was separated by SDS-PAGE using a 10% gel and blotted onto nitrocellulose membranes (Hybond ECL, Amersham Biosciences, Piscataway, NJ, USA). Membranes were blocked by incubation in Tris-buffered saline (TBS) containing 5% non-fat dry milk and 0.2% Tween 20, for 1 h at room temperature, and were then incubated overnight at 4° C with primary rabbit monoclonal anti-human HOXA5 antibody from Abcam (1:500, Cat No. ab140636, Abcam, Cambridge, UK) and primary rabbit monoclonal anti-human PTEN antibody from Abcam (1:1000, Cat No. ab32199, Abcam, Cambridge, UK) respectively. Blots were washed and incubated at room temperature for 1 h with IRDye 800CW goat anti-rabbit IgG secondary antibody (diluted 1:15,000, Cat No. 926-32211, LI-COR, Lincoln, Nebraska USA). Bands were visualized by LI-COR Odyssey CLx imaging system (LI-COR). ß-actin served as a loading control (mouse monoclonal anti-human ß-actin, 1:51:1000, Cat No. ab8226, Abcam; IRDye 680RD goat anti-mouse secondary antibody, Cat No. 926-68070, LI-COR).

### Calculations and statistical methods

Methylation and expression profiles are shown as mean and standard error of the mean (SEM). The statistical significance of differences between groups was determined by the Student´s t test. The significance levels were P < 0.05, P < 0.01 or P < 0.001, respectively.

## Supplementary Material

Supplementary Table 1

Supplementary Table 2
