# Supplementary material for: An aging and p53 related marker: HOXA5 promoter methylation negatively correlates with mRNA and protein expression in old age
Source: Aging (Albany NY). 2021 Feb 5;13(4):4831–49. doi: 10.18632/aging.202621 (PMC7950283; doi:10.18632/aging.202621)
Supplement: Supplementary Table 1 [file aging-13-202621-s001.doc]

|  | Target CpGs investigated here | |
| --- | --- | --- |
|  | Overlap with RnBeads pipeline | |
|  | Overlap with Horvath's clock | |
|  | Overlap with both data sets | |
|  |  |  |
| cgid | Chromosome | annotation |
| cg09780150 | chr18 | CABLES1,CABLES1,CABLES1,CABLES1/Body,Body,Body,Body/- |
| cg21139150 | chr21 | -/-/Island |
| cg16361921 | chr5 | -/-/- |
| cg07158505 | chr18 | -/-/- |
| cg03192186 | chr7 | -/-/- |
| cg20004147 | chr2 | -/-/- |
| cg10511229 | chr20 | -/-/- |
| cg24125828 | chr6 | PRRT1/Body/Island |
| cg22454769 | chr2 | FHL2,FHL2,FHL2,FHL2/TSS200,TSS200,5'UTR,TSS200/Island |
| cg06856169 | chr5 | -/-/- |
| cg13108341 | chr17 | DNAH9/Body/- |
| cg25340709 | chr20 | RRBP1,RRBP1/Body,Body/S_Shore |
| cg04876534 | chr4 | CLRN2/Body/S_Shelf |
| cg07971513 | chr8 | EXTL3,EXTL3,EXTL3/Body,Body,Body/- |
| cg10444583 | chr16 | NOD2,NOD2/5'UTR,1stExon/- |
| cg26893861 | chr17 | DUSP3/3'UTR/- |
| cg25751474 | chr14 | -/-/- |
| cg04547000 | chr16 | -/-/- |
| cg01256539 | chr5 | PRR16/5'UTR/S_Shore |
| cg07553761 | chr3 | TRIM59/TSS1500/Island |
| cg11847992 | chr5 | -/-/- |
| cg14021929 | chr7 | TYW1/Body/- |
| cg06639320 | chr2 | FHL2,FHL2,FHL2,FHL2/TSS200,TSS200,5'UTR,TSS200/Island |
| cg27520474 | chr16 | CES1,CES1,CES1/TSS1500,TSS1500,TSS1500/S_Shore |
| cg21184711 | chr7 | CADPS2,CADPS2,CADPS2/Body,Body,Body/- |
| cg24969716 | chr11 | LOC399886/Body/- |
| cg09417038 | chr21 | C21orf57/Body/N_Shore |
| cg03744763 | chr7 | HOXA5/TSS1500/Island |
| cg03792936 | chr2 | CCDC148,CCDC148/Body,Body/- |
| cg16532938 | chr2 | FIGN/Body/- |
| cg03876418 | chr11 | -/-/- |
| cg10834425 | chr12 | INHBC/Body/- |
| cg07909165 | chr6 | CD164,CD164,CD164,CD164,CD164/Body,Body,Body,Body,Body/- |
| cg09732868 | chr4 | COX7B2/5'UTR/- |
| cg21358336 | chr17 | -/-/Island |
| cg23174406 | chr12 | NEDD1,NEDD1,NEDD1,NEDD1/Body,Body,Body,5'UTR/S_Shelf |
| cg07183775 | chr7 | -/-/N_Shelf |
| cg10243322 | chr19 | KDM4B/Body/N_Shelf |
| cg06526721 | chr5 | PRR16/TSS1500/N_Shore |
| cg02140579 | chr4 | FAM198B,FAM198B,FAM198B/Body,Body,Body/- |
| cg11128862 | chr1 | SOX13/5'UTR/- |
| cg17268658 | chr2 | FHL2,FHL2,FHL2,FHL2/TSS200,TSS200,TSS200,5'UTR/Island |
| cg18645241 | chr21 | -/-/- |
| cg21758126 | chr2 | NR4A2/Body/N_Shore |
| cg11883129 | chr2 | FOXN2/5'UTR/- |
| cg14222729 | chr2 | -/-/N_Shore |
| cg14886849 | chr14 | TRAF3,TRAF3,TRAF3/Body,Body,Body/- |
| cg06090322 | chr3 | -/-/- |
| cg06895752 | chr14 | -/-/- |
| cg02753903 | chr19 | RYR1,RYR1/Body,Body/Island |
| cg02872426 | chr6 | DDO,DDO/TSS200,TSS200/- |
| cg11236452 | chr5 | PCDHGA2,PCDHGA3,PCDHGA3,PCDHGA1/Body,1stExon,1stExon,Body/N_Shore |
| cg14679668 | chr8 | -/-/- |
| cg02008229 | chr15 | ANPEP/Body/- |
| cg02486239 | chr1 | -/-/- |
| cg27262717 | chr20 | TNNC2/Body/Island |
| cg21770758 | chr10 | RBM20/Body/- |
| cg27245231 | chr10 | GHITM/TSS1500/N_Shore |
| cg23589035 | chr1 | ASH1L/Body/- |
| cg07112541 | chr13 | -/-/N_Shore |
| cg04875128 | chr15 | OTUD7A/Body/Island |
| cg10920224 | chr14 | TRAF3,TRAF3,TRAF3/Body,Body,Body/- |
| cg16789844 | chr7 | PDE1C/TSS200/S_Shore |
| cg19344626 | chr19 | NWD1/TSS200/- |
| cg13771313 | chr11 | ATG16L2/Body/Island |
| cg24707573 | chr7 | SH2B2/Body/Island |
| cg24049493 | chr1 | HIVEP3,HIVEP3/TSS1500,TSS1500/S_Shore |
| cg21764456 | chr16 | TEKT5/Body/- |
| cg21700663 | chr15 | -/-/N_Shore |
| cg24079051 | chr2 | LOC643387,LOC151174,LOC151174/Body,TSS200,TSS200/Island |
| cg23080527 | chr17 | DUSP3/3'UTR/- |
| cg13782301 | chr6 | PRRT1/3'UTR/Island |
| cg01516851 | chr6 | -/-/- |
| cg06344992 | chr15 | ANPEP/Body/- |
| cg11071401 | chr17 | CACNA1G,CACNA1G,CACNA1G,CACNA1G,CACNA1G,CACNA1G,CACNA1G,CACNA1G,CACNA1G,CACNA1G,CACNA1G,CACNA1G,CACNA1G,CACNA1G,CACNA1G/TSS1500,TSS1500,TSS1500,TSS1500,TSS1500,TSS1500,TSS1500,TSS1500,TSS1500,TSS1500,TSS1500,TSS1500,TSS1500,TSS1500,TSS1500/Island |
| cg20118553 | chr13 | STARD13,STARD13,STARD13,STARD13/5'UTR,5'UTR,Body,Body/- |
| cg04402345 | chr5 | -/-/- |
| cg21531089 | chr1 | STPG1,STPG1,STPG1,STPG1/Body,Body,Body,5'UTR/- |
| cg16579158 | chr5 | PCDHGA2,PCDHGA1,PCDHGA2/1stExon,Body,1stExon/Island |
| cg15477247 | chr5 | -/-/Island |
| cg26332926 | chr10 | PRKG1,PRKG1/Body,Body/S_Shore |
| cg19570618 | chr3 | BBX,BBX,BBX/5'UTR,5'UTR,5'UTR/- |
| cg27445894 | chr17 | -/-/- |
| cg24892069 | chr10 | NRP1,NRP1,NRP1/Body,Body,Body/- |
| cg26077615 | chr11 | PPFIA1,PPFIA1,PPFIA1/Body,Body,Body/- |
| cg08997126 | chr1 | -/-/- |
| cg04455146 | chr7 | CADPS2,CADPS2,CADPS2/Body,Body,Body/- |
| cg04252928 | chr16 | ACSF3,ACSF3,ACSF3/Body,Body,Body/S_Shore |
| cg02910018 | chr12 | TMTC1,TMTC1/Body,Body/- |
| cg09124496 | chr7 | LOC285954,LOC285954,INHBA/Body,Body,Body/- |
| cg12346592 | chr13 | -/-/Island |
| cg22329875 | chr8 | -/-/- |
| cg03650189 | chr19 | ICAM5/Body/Island |
| cg19151852 | chr17 | ASPSCR1,ASPSCR1,ASPSCR1/Body,Body,Body/S_Shelf |
| cg13574913 | chr10 | ZMIZ1/Body/- |
| cg16769658 | chr1 | MAEL,MAEL,MAEL/Body,Body,Body/- |
| cg24690094 | chr11 | -/-/Island |
| cg24869272 | chr11 | TSPAN4,TSPAN4,TSPAN4,TSPAN4,TSPAN4,/5'UTR,5'UTR,5'UTR,5'UTR,5'UTR,/Island |
| cg19663246 | chr7 | -/-/S_Shore |
| cg09359064 | chr9 | NTNG2/5'UTR/Island |
| cg08952306 | chr7 | SH2B2/3'UTR/Island |
| cg15852440 | chr6 | DNAH8/Body/- |
| cg02846841 | chr6 | -/-/- |
| cg27380997 | chr15 | -/-/S_Shore |
| cg03468072 | chr12 | -/-/Island |
| cg12080266 | chr21 | CLIC6/Body/- |
| cg27142377 | chr12 | -/-/- |
| cg22805485 | chr11 | SPON1/TSS200/N_Shore |
| cg05130482 | chr8 | -/-/- |
| cg15417294 | chr10 | SLC18A2/Body/- |
| cg04352272 | chr17 | -/-/Island |
| cg05217193 | chr22 | DENND6B,DENND6B/ExonBnd,Body/- |
| cg25410668 | chr1 | RPA2/TSS1500/S_Shore |
| cg09137095 | chr5 | -/-/- |
| cg23972165 | chr2 | -/-/- |
| cg19873923 | chr14 | TC2N,TC2N,TC2N/5'UTR,5'UTR,5'UTR/N_Shore |
| cg10089963 | chr2 | NR4A2/Body/N_Shore |
| cg17113038 | chr5 | PRR16,PRR16,PRR16,PRR16/Body,5'UTR,Body,5'UTR/- |
| cg16538682 | chr7 | CADPS2,CADPS2,CADPS2/Body,Body,Body/- |
| cg19317830 | chr3 | VGLL4,VGLL4,VGLL4/Body,Body,Body/- |
| cg03052078 | chr6 | STXBP5,STXBP5/Body,Body/S_Shore |
| cg20398091 | chr19 | NWD1,NWD1/TSS1500,TSS1500/- |
| cg12256845 | chr6 | PTCHD4,PTCHD4/TSS1500,TSS1500/S_Shore |
| cg12158483 | chr22 | BAIAP2L2/Body/Island |
| cg10919664 | chr6 | -/-/- |
| cg18345561 | chr15 | -/-/- |
| cg17035899 | chr4 | MYL5/Body/Island |
| cg24102222 | chr12 | SLC15A4/Body/Island |
| cg11204139 | chr17 | -/-/Island |
| cg04559928 | chr7 | LOC100630923,PRKRIP1/Body,Body/- |
| cg27151303 | chr7 | -/-/Island |
| cg14812628 | chr19 | -/-/- |
| cg17514249 | chr13 | COL4A1,COL4A1/Body,Body/- |
| cg12031275 | chr15 | -/-/N_Shore |
| cg03338962 | chr14 | KIAA0391,KIAA0391,KIAA0391,KIAA0391,KIAA0391/Body,Body,Body,Body,Body/- |
| cg03807316 | chr19 | DLL3,DLL3/Body,Body/Island |
| cg13721246 | chr12 | DNAH10/Body/- |
| cg09394785 | chr11 | ASCL2/TSS1500/S_Shore |
| cg12800028 | chr6 | GPR6/TSS200/Island |
| cg03324695 | chr1 | -/-/- |
| cg01088900 | chr8 | ST3GAL1,ST3GAL1/5'UTR,5'UTR/- |
| cg01265905 | chr20 | -/-/- |
| cg16505764 | chr5 | PCDHGA4,PCDHGA7,PCDHGA6,PCDHGA1,PCDHGA5/Body,1stExon,Body,Body,Body/N_Shore |
| cg19439768 | chr7 | REPIN1,REPIN1,REPIN1,REPIN1,REPIN1/1stExon,3'UTR,3'UTR,3'UTR,3'UTR/S_Shore |
| cg02048733 | chr3 | -/-/- |
| cg11084334 | chr3 | LHFPL4/Body/Island |
| cg17293161 | chr10 | -/-/- |
| cg06291867 | chr10 | HTR7,HTR7,HTR7/1stExon,1stExon,1stExon/Island |
| cg25258740 | chr12 | KCNC2,KCNC2,KCNC2,KCNC2,KCNC2,KCNC2/5'UTR,5'UTR,5'UTR,5'UTR,5'UTR,5'UTR/Island |
| cg07910479 | chr6 | LY86,LY86-AS1/TSS1500,Body/- |
| cg07547549 | chr20 | SLC12A5,SLC12A5/Body,Body/Island |
| cg08551532 | chr19 | DLL3,DLL3/Body,Body/Island |
| cg16658255 | chr5 | -/-/N_Shelf |
| cg05542681 | chr16 | FBXL16/Body/Island |
| cg12841266 | chr3 | LHFPL4/Body/Island |
| cg00563701 | chr18 | -/-/- |
| cg21862353 | chr2 | MYT1L/Body/Island |
| cg09936799 | chr5 | PCDHA6,PCDHA2,PCDHA1,PCDHA9,PCDHA7,PCDHA1/Body,Body,Body,Body,Body,Body/N_Shore |
| cg27398495 | chr3 | -/-/- |
| cg23758309 | chr12 | -/-/- |
| cg00329615 | chr3 | IGSF11,IGSF11/Body,Body/- |
| cg05767720 | chr6 | PRRT1/Body/N_Shore |
| cg01748892 | chr7 | HOXA5/TSS1500/Island |
| cg15922174 | chr9 | CRB2/Body/Island |
| cg16464373 | chr8 | -/-/S_Shelf |
| cg17110586 | chr19 | -/-/S_Shelf |
| cg13694927 | chr7 | HOXA5/TSS1500/Island |
| cg08264839 | chr11 | -/-/- |
| cg03162326 | chr6 | SOBP/Body/Island |
| cg06375806 | chr22 | DENND6B,DENND6B/ExonBnd,Body/- |
| cg08057120 | chr11 | -/-/- |
| cg06975979 | chr7 | LRRC61,ACTR3C,LRRC61/TSS1500,5'UTR,TSS1500/Island |
| cg16627090 | chr7 | PGAM2/1stExon/Island |
| cg03032497 | chr14 | -/-/N_Shore |
| cg17391820 | chr16 | -/-/N_Shore |
| cg27601844 | chr18 | SKOR2,SKOR2/TSS200,TSS200/S_Shore |
| cg21159778 | chr9 | DFNB31,DFNB31/TSS1500,1stExon/Island |
| cg04193160 | chr1 | OBSCN,OBSCN/Body,Body/Island |
| cg15167429 | chr10 | -/-/Island |
| cg05573844 | chr12 | -/-/Island |
| cg14481208 | chr2 | RTKN/TSS1500/Island |
| cg15011409 | chr19 | ICAM5/Body/Island |
| cg05179035 | chr3 | -/-/N_Shore |
| cg14798125 | chr1 | GPR52,RABGAP1L/1stExon,Body/- |
| cg11876952 | chr12 | -/-/- |
| cg16574737 | chr5 | PCDHGB1,PCDHGA2,PCDHGA1,PCDHGB1,PCDHGA3/1stExon,Body,Body,1stExon,Body/N_Shore |
| cg01679206 | chr7 | WBSCR27/Body/N_Shelf |
| cg17438696 | chr1 | -/-/- |
| cg15773296 | chr14 | SYNE2,SYNE2,MIR548AZ/Body,Body,Body/- |
| cg10012273 | chr5 | PDE4D,PDE4D,PDE4D,PDE4D,PDE4D,PDE4D/TSS1500,Body,Body,Body,Body,Body/- |
| cg07164639 | chr6 | DDO,DDO/TSS1500,TSS1500/- |
| cg15549544 | chr15 | -/-/- |
| cg15046693 | chr19 | CEBPG/TSS1500/N_Shore |
| cg06532574 | chr5 | ELL2/Body/N_Shore |
| cg24101670 | chr5 | PCDHGB3,PCDHGB3,PCDHGA1,PCDHGA2,PCDHGA3/TSS1500,TSS1500,Body,Body,Body/N_Shore |
| cg19935040 | chr17 | FAM171A2/Body/Island |
| cg02016419 | chr17 | TEKT3/TSS1500/S_Shore |
| cg03764753 | chr2 | -/-/- |
| cg11291003 | chr5 | -/-/N_Shore |
| cg09366265 | chr17 | EZH1/Body/- |
| cg15672768 | chr5 | PCDHGA4,PCDHGA2,PCDHGB2,PCDHGA1,PCDHGB1/Body,Body,1stExon,Body,Body/Island |
| cg21570988 | chr6 | PRRT1/3'UTR/Island |
| cg08296461 | chr9 | MVB12B/Body/- |
| cg25478614 | chr3 | SST/Body/N_Shore |
| cg23825213 | chr18 | KCNG2/TSS200/Island |
| cg06527016 | chr16 | CES1,CES1,CES1/Body,Body,Body/- |
| cg16164607 | chr1 | -/-/- |
| cg02282631 | chr5 | -/-/S_Shore |
| cg08076830 | chr18 | -/-/Island |
| cg07584066 | chr17 | DHX40,DHX40/TSS200,TSS200/Island |
| cg14498475 | chr8 | LY6K,LY6K,LY6K,LY6K,LY6K,LY6K/5'UTR,5'UTR,5'UTR,1stExon,1stExon,1stExon/Island |
| cg23149687 | chr5 | PRR16/5'UTR/S_Shore |
| cg05144700 | chr6 | -/-/- |
| cg03481131 | chr9 | DFNB31,DFNB31,DFNB31/TSS1500,1stExon,1stExon/Island |
| cg05003599 | chr7 | FBXO24,FBXO24,FBXO24/Body,1stExon,Body/S_Shelf |
| cg20938085 | chr5 | PCDHGB4,PCDHGA4,PCDHGA6,PCDHGA1,PCDHGA5/1stExon,Body,Body,Body,Body,/N_Shore |
| cg20665157 | chr7 | CADPS2,CADPS2,CADPS2/Body,Body,Body/- |
| cg25903783 | chr17 | -/-/Island |
| cg18158739 | chr14 | -/-/- |
| cg00199091 | chr6 | TRERF1/Body/S_Shore |
| cg24866418 | chr3 | LHFPL4/Body/Island |
| cg21044139 | chr16 | PKD1L2,PKD1L2,PKD1L2/TSS200,TSS200,TSS200/- |
| cg22987448 | chr19 | MYO1F/Body/Island |
| cg05365033 | chr19 | CRTC1,CRTC1/3'UTR,3'UTR/S_Shore |
| cg10184289 | chr2 | SERTAD2/5'UTR/- |
| cg24716530 | chr11 | LTBP3,LTBP3,LTBP3/Body,Body,Body/Island |
| cg12357115 | chr8 | FAM84B/Body/Island |
| cg26612727 | chr17 | ZPBP2,ZPBP2/1stExon,1stExon/Island |
| cg03483626 | chr1 | KCNA3/TSS1500/S_Shore |
| cg26947034 | chr7 | -/-/- |
| cg21572722 | chr6 | ELOVL2/TSS1500/Island |
| cg26133418 | chr1 | -/-/- |
| cg09310092 | chr19 | SCN1B/Body/N_Shelf |
| cg25516803 | chr3 | CCK/TSS1500/Island |
| cg00787180 | chr14 | CCDC88C/Body/N_Shelf |
| cg20591472 | chr1 | SYPL2/TSS200/Island |
| cg16966496 | chr12 | -/-/N_Shore |
| cg08754025 | chr7 | PDE1C/TSS200/S_Shore |
| cg09118625 | chr1 | DIRAS3/Body/Island |
| cg16307919 | chr19 | ZNF428/Body/- |
| cg06279276 | chr16 | B3GNT9/Body/Island |
| cg23188684 | chr11 | -/-/Island |
| cg09372060 | chr7 | PDE1C/TSS200/S_Shore |
| cg10323433 | chr13 | HTR2A,HTR2A/TSS1500,TSS1500/- |
| cg21915313 | chr5 | PCDHGA4,PCDHGA6,PCDHGA1,PCDHGA5,PCDHGB1/Body,Body,Body,Body,Body/Island |
| cg14637125 | chr12 | -/-/- |
| cg06891424 | chr19 | RYR1,RYR1,RYR1,RYR1/ExonBnd,ExonBnd,Body,Body/- |
| cg25800500 | chr2 | ANKRD23/3'UTR/Island |
| cg05772939 | chr2 | LOC101927053,FAM178B/Body,Body/- |
| cg08796299 | chr5 | PRR16,PRR16,PRR16/Body,5'UTR,Body/- |
| cg11970349 | chr4 | GPR78/TSS200/Island |
| cg15367253 | chr19 | SNAR-G2,CGB2/Body,TSS200/- |
| cg10478315 | chr19 | -/-/- |
| cg18269963 | chr4 | -/-/N_Shore |
| cg14383544 | chr11 | KCNJ5/5'UTR/S_Shelf |
| cg25003531 | chr12 | KSR2/Body/- |
| cg25573114 | chr2 | RTKN/TSS1500/S_Shore |
| cg19192159 | chr5 | PCDHGA5,PCDHGA5,PCDHGA1,PCDHGA2/TSS200,TSS200,Body,Body/N_Shore |
| cg11945372 | chr22 | MICAL3,MICAL3,MICAL3/TSS1500,TSS1500,TSS1500/S_Shore |
| cg18848419 | chr18 | CHMP1B,GNAL,GNAL,GNAL/TSS1500,Body,Body,Body/N_Shore |
| cg18468088 | chr6 | -/-/- |
| cg13085024 | chr16 | -/-/- |
| cg07323488 | chr3 | EGFEM1P/Body/- |
| cg00151370 | chr6 | ATXN1,ATXN1/Body,Body/N_Shelf |
| cg17560061 | chr1 | -/-/S_Shore |
| cg21165089 | chr11 | C11orf85,C11orf85/TSS200,TSS200/Island |
| cg05993265 | chr4 | MFSD10,MFSD10/Body,Body/Island |
| cg06336535 | chr19 | FCHO1,FCHO1,FCHO1,FCHO1/Body,Body,Body,Body/Island |
| cg25645064 | chr3 | -/-/- |
| cg17090409 | chr11 | CHRDL2,CHRDL2/TSS1500,TSS1500/S_Shore |
| cg01557798 | chr1 | OBSCN,OBSCN/Body,Body/Island |
| cg00059225 | chr5 | GLRA1,GLRA1,GLRA1,GLRA1/1stExon,1stExon,5'UTR,5'UTR/Island |
| cg20249566 | chr19 | NWD1/TSS200/- |
| cg15030397 | chr5 | PCDHGA4,PCDHGA7,PCDHGA6,PCDHGA1,PCDHGA5/Body,1stExon,Body,Body,Body/Island |
| cg00979931 | chr11 | ATG16L2/Body/Island |
| cg10832308 | chr13 | ENOX1,ENOX1,ENOX1/Body,Body,Body/- |
| cg20461188 | chr10 | -/-/Island |
| cg22818074 | chr15 | MAP2K1/Body/- |
| cg06563300 | chr12 | SLC17A8,SLC17A8/TSS200,TSS200/- |
| cg18145105 | chr2 | NEURL3/Body/Island |
| cg18071806 | chr12 | AEBP2,AEBP2,AEBP2/TSS1500,TSS1500,TSS1500/N_Shore |
| cg06853416 | chr1 | -/-/S_Shore |
| cg11935615 | chr16 | KIAA0430,KIAA0430,KIAA0430/Body,Body,Body/- |
| cg24878173 | chr11 | ATG16L2/Body/Island |
| cg24147779 | chr15 | CRTC3,CRTC3/Body,Body/- |
| cg11980781 | chr14 | -/-/- |
| cg00571190 | chr20 | HNF4A,HNF4A,HNF4A,HNF4A,HNF4A/TSS1500,TSS1500,TSS1500,TSS1500,TSS1500/- |
| cg24365804 | chr19 | TTYH1,TTYH1,TTYH1/Body,Body,Body/- |
| cg20248866 | chr17 | PCYT2/TSS1500/Island |
| cg20660424 | chr22 | TTLL12/Body/N_Shelf |
| cg07231986 | chr15 | -/-/- |
| cg03465894 | chr11 | -/-/- |
| cg22901840 | chr1 | DIRAS3/Body/Island |
| cg06677190 | chr19 | ONECUT3/Body/S_Shore |
| cg10243278 | chr14 | OTX2,OTX2,OTX2,OTX2/TSS200,TSS200,TSS200,TSS200/S_Shore |
| cg03461110 | chr7 | FOXK1/Body/- |
| cg17621438 | chr5 | RNF180,RNF180/TSS1500,TSS1500/N_Shore |
| cg01579024 | chr5 | RANBP17/TSS1500/N_Shore |
| cg13327545 | chr10 | -/-/Island |
| cg08184159 | chr12 | TBX5,TBX5,TBX5,TBX5/Body,Body,Body,Body/N_Shore |
| cg23462697 | chr12 | -/-/N_Shelf |
| cg07703654 | chr8 | GOLSYN,GOLSYN/TSS1500,TSS1500/S_Shore |
| cg11734017 | chr1 | RHD,RHD,RHD,RHD,RHD,RHD,RHD,RHD/5'UTR,Body,Body,Body,Body,Body,Body,Body/- |
| cg12957265 | chr15 | ZNF280D,ZNF280D,ZNF280D/TSS1500,TSS1500,TSS1500/Island |
| cg14522990 | chr6 | C6orf25,C6orf25,C6orf25,C6orf25,C6orf25,C6orf25/Body,Body,Body,Body,Body,Body/Island |
| cg00362690 | chr15 | MIR548H4/Body/- |
| cg26962778 | chr5 | PCDHGA4,PCDHGA2,PCDHGB2,PCDHGA1,PCDHGB1/Body,Body,1stExon,Body,Body/N_Shore |
| cg07442271 | chr5 | PCDHGA2,PCDHGA4,PCDHGA1,PCDHGB1,PCDHGA3/Body,1stExon,Body,Body,Body/N_Shore |
| cg22897615 | chr6 | PRRT1/Body/Island |
| cg21487550 | chr3 | -/-/Island |
| cg00958560 | chr4 | C4orf50/Body/- |
| cg14620941 | chr9 | -/-/N_Shelf |
| cg26394870 | chr1 | LOC102724312,LOC102724312,LOC102724312/Body,Body,Body/N_Shelf |
| cg11372636 | chr6 | GPR6,GPR6,GPR6,GPR6/1stExon,1stExon,5'UTR,5'UTR/Island |
| cg25345365 | chr11 | ZBTB16,ZBTB16/Body,Body/- |
| cg01542019 | chr19 | TECR/Body/N_Shelf |
| cg00481259 | chr16 | DECR2/TSS1500/Island |
| cg24213248 | chr2 | AGAP1,AGAP1/Body,Body/- |
| cg07476379 | chr3 | SOX2-OT,SOX2-OT,SOX2-OT/Body,Body,Body/- |
| cg21911021 | chr19 | ZIK1/TSS1500/Island |
| cg17473341 | chr12 | USP44,USP44,USP44/5'UTR,5'UTR,5'UTR/N_Shore |
| cg05339962 | chr2 | -/-/- |
| cg15089111 | chr14 | NPAS3,NPAS3,NPAS3,NPAS3/Body,Body,Body,Body/Island |
| cg19398269 | chr6 | -/-/- |
| cg15190383 | chr19 | CRTC1,CRTC1/3'UTR,3'UTR/Island |
| cg17434842 | chr10 | -/-/- |
| cg25423428 | chr22 | SULT4A1/Body/N_Shelf |
| cg18746443 | chr5 | LOC101929710/Body/- |
| cg14996263 | chr12 | ATN1,ATN1/Body,Body/Island |
| cg10180092 | chr3 | TNIK,TNIK,TNIK,TNIK,TNIK,TNIK,TNIK,TNIK/Body,Body,Body,Body,Body,Body,Body,Body/- |
| cg15084543 | chr1 | ELTD1,ELTD1/5'UTR,1stExon/Island |
| cg10313047 | chr3 | DNAH1/5'UTR/- |
| cg12662084 | chr6 | KIF13A,KIF13A,KIF13A,KIF13A/Body,Body,Body,Body/- |
| cg27459131 | chr3 | P3H2,P3H2/Body,5'UTR/- |
| cg23752752 | chr7 | FOXK1/Body/- |
| cg17689735 | chr8 | SGCZ/TSS200/S_Shore |
| cg27067781 | chr6 | PRRT1/3'UTR/Island |
| cg06435901 | chr11 | OPCML/Body/- |
| cg03845654 | chr2 | FMNL2/Body/- |
| cg08662753 | chr9 | COL5A1,COL5A1/Body,Body/- |
| cg11847597 | chr8 | PEBP4/Body/- |
| cg14767165 | chr6 | ACAT2/Body/S_Shore |
| cg24265195 | chr2 | DYSF,DYSF,DYSF,DYSF,DYSF,DYSF,DYSF,DYSF/Body,Body,Body,Body,Body,Body,Body,Body/- |
| cg02428792 | chr4 | -/-/S_Shore |
| cg13696752 | chr9 | C9orf68,PPAPDC2/Body,1stExon/Island |
| cg11174855 | chr10 | NKX6-2/3'UTR/Island |
| cg26239485 | chr2 | AGAP1,AGAP1/Body,Body/- |
| cg27552418 | chr4 | -/-/- |
| cg08855133 | chr1 | HIVEP3/Body/- |
| cg08254359 | chr12 | -/-/N_Shore |
| cg06335143 | chr1 | ZYG11A/Body/Island |
| cg02624047 | chr2 | STON1,STON1,STON1-GTF2A1L/TSS1500,TSS1500,TSS200/N_Shore |
| cg12902206 | chr3 | DNAH1/5'UTR/- |
| cg00633552 | chr5 | PCDHGA4,PCDHGA2,PCDHGB2,PCDHGA1/Body,Body,1stExon,Body/N_Shore |
| cg05025860 | chr4 | -/-/- |
| cg09526429 | chr4 | -/-/- |
| cg21003654 | chr4 | JAKMIP1,JAKMIP1/Body,Body/Island |
| cg12598870 | chr11 | NELL1,NELL1/Body,Body/- |
| cg15907944 | chr22 | -/-/Island |
| cg14696217 | chr22 | -/-/- |
| cg11323285 | chr12 | TRHDE-AS1,TRHDE-AS1/Body,Body/N_Shelf |
| cg00513205 | chr5 | NEUROG1/TSS1500/S_Shore |
| cg02188818 | chr4 | TBC1D9/Body/N_Shore |
| cg04906447 | chr1 | SLC35F3,SLC35F3/Body,Body/- |
| cg02605776 | chr1 | C1orf103,C1orf103/TSS1500,TSS1500/S_Shore |
| cg01369611 | chr5 | LOC101929710/Body/- |
| cg02063488 | chr2 | FBXO41/Body/Island |
| cg04656321 | chr19 | LOC100128398,LOC100128398,ZNF606/Body,ExonBnd,TSS200/S_Shore |
| cg25264181 | chr8 | OXR1,OXR1,OXR1/Body,Body,Body/- |
| cg08637691 | chr9 | -/-/- |
| cg14003022 | chr4 | -/-/Island |
| cg04427498 | chr7 | -/-/Island |
| cg01719405 | chr14 | -/-/- |
| cg06540876 | chr6 | ZBTB12/TSS1500/S_Shore |
| cg11705975 | chr10 | PRLHR/Body/Island |
| cg17885226 | chr6 | -/-/Island |
| cg03095642 | chr1 | SLC5A9,SLC5A9/Body,Body/- |
| cg08243356 | chr18 | CTIF,CTIF/Body,Body/- |
| cg01763090 | chr15 | OTUD7A/3'UTR/N_Shore |
| cg02194492 | chr10 | MAT1A,MAT1A/1stExon,5'UTR/- |
| cg17208001 | chr18 | PIEZO2/Body/- |
| cg23614947 | chr14 | PSMC6/TSS1500/N_Shore |
| cg20149168 | chr1 | DIRAS3/Body/Island |
| cg06448705 | chr5 | TRPC7,TRPC7,TRPC7,TRPC7,TRPC7,TRPC7/5'UTR,1stExon,1stExon,1stExon,5'UTR,5'UTR/- |
| cg14951955 | chr6 | ATG5/TSS1500/S_Shore |
| cg25936138 | chr5 | -/-/- |
| cg00921839 | chr1 | -/-/- |
| cg16362203 | chr18 | BOD1L2/TSS200/- |
| cg14556683 | chr19 | EPHX3,EPHX3/1stExon,Body/Island |
| cg19029904 | chr13 | -/-/- |
| cg01207684 | chr16 | ADCY9/Body/- |
| cg04503319 | chr16 | ANKRD11/Body/N_Shelf |
| cg26290632 | chr8 | CALB1/1stExon/- |
| cg03047995 | chr14 | FITM1/Body/Island |
| cg13935689 | chr1 | -/-/S_Shore |
| cg07893565 | chr4 | HPSE,HPSE,HPSE,HPSE/Body,Body,Body,Body/- |
| cg05622025 | chr6 | -/-/- |
| cg25430089 | chr9 | PBX3,PBX3,PBX3,PBX3/Body,Body,Body,Body/- |
| cg13696706 | chr9 | DAB2IP/Body/- |
| cg14209133 | chr5 | PCDHGA4,PCDHGA1,PCDHGA6,PCDHGA5,PCDHGB1/Body,Body,1stExon,Body,Body/Island |
| cg06399965 | chr16 | -/-/- |
| cg14255824 | chr9 | TJP2,TJP2,TJP2,TJP2/Body,5'UTR,Body,Body/- |
| cg01410876 | chr19 | LMTK3/Body/Island |
| cg01028526 | chr4 | TMPRSS11BNL/TSS1500/- |
| cg13552692 | chr18 | CCDC102B/5'UTR/- |
| cg00481951 | chr3 | SST/Body/N_Shore |
| cg26670674 | chr14 | -/-/N_Shore |
| cg00663642 | chr13 | -/-/- |
| cg26234795 | chr6 | -/-/S_Shelf |
| cg22138393 | chr8 | PTDSS1/Body/- |
| cg01011173 | chr2 | -/-/- |
| cg23174607 | chr6 | PTP4A1/TSS200/N_Shore |
| cg18259003 | chr17 | KIF19/Body/Island |
| cg06784991 | chr1 | ZYG11A/Body/Island |
| cg22358580 | chr12 | HOXC4,HOXC5,HOXC5/5'UTR,TSS200,Body/N_Shore |
| cg07930159 | chr8 | TPD52,TPD52,TPD52,TPD52,TPD52,TPD52,TPD52/Body,Body,Body,Body,Body,Body,Body/- |
| cg24579753 | chr13 | MTUS2,MTUS2/Body,Body/- |
| cg18738190 | chr10 | CHST3/5'UTR/- |
| cg08586168 | chr3 | ADCY5/TSS1500/Island |
| cg12910087 | chr4 | -/-/- |
| cg06601581 | chr16 | -/-/N_Shelf |
| cg08119153 | chr14 | NRXN3,NRXN3/5'UTR,Body/- |
| cg11376375 | chr1 | SUSD4/Body/- |
| cg23181554 | chr10 | -/-/Island |
| cg20544848 | chr19 | ELAVL3,ELAVL3/TSS200,TSS200/Island |
| cg10211414 | chr1 | RERE,RERE/5'UTR,5'UTR/N_Shore |
| cg21599943 | chr17 | -/-/- |
| cg07991335 | chr10 | WDR11-AS1,MIR5694/Body,Body/- |
| cg12534424 | chr7 | PRRT4/Body/Island |
| cg00366917 | chr18 | MBP,MBP/TSS1500,TSS1500/Island |
| cg20366299 | chr3 | DALRD3,DALRD3,DALRD3,NDUFAF3/5'UTR,TSS1500,TSS1500,TSS1500/N_Shore |
| cg06648759 | chr13 | -/-/- |
| cg19784428 | chr19 | NWD1/TSS200/- |
| cg06726154 | chr8 | -/-/- |
| cg01820962 | chr6 | NT5DC1/Body/- |
| cg11954763 | chr3 | TMEM108,TMEM108,TMEM108/5'UTR,5'UTR,5'UTR/- |
| cg05768419 | chr1 | GPR52,RABGAP1L/1stExon,Body/- |
| cg04004558 | chr16 | SOCS1/3'UTR/Island |
| cg20774052 | chr7 | LOC100505921,GLCCI1/TSS1500,Body/S_Shore |
| cg11564711 | chr18 | -/-/Island |
| cg10380546 | chr11 | AP2A2,AP2A2/Body,Body/N_Shore |
| cg14179389 | chr1 | GFI1,GFI1,GFI1/Body,Body,Body/Island |
| cg19136485 | chr14 | KIAA0125/Body/- |
| cg27057509 | chr6 | VARS2,VARS2,VARS2/Body,Body,Body/S_Shore |
| cg23294940 | chr10 | ANAPC16,ANAPC16,ANAPC16,ANAPC16,ASCC1/5'UTR,5'UTR,5'UTR,5'UTR,TSS1500/S_Shore |
| cg19419291 | chr5 | ELL2/Body/N_Shore |
| cg09126156 | chr9 | STXBP1,STXBP1/3'UTR,Body/N_Shelf |
| cg12590902 | chr1 | ERI3/Body/- |
| cg17592231 | chr11 | -/-/S_Shore |
| cg18501647 | chr6 | PRRT1/Body/N_Shore |
| cg25174343 | chr21 | RIPK4/Body/N_Shore |
| cg04265051 | chr11 | LRP5/TSS1500/N_Shore |
| cg14961794 | chr11 | OR9Q1/5'UTR/- |
| cg22099081 | chr12 | -/-/- |
| cg19135247 | chr21 | SIK1/Body/S_Shelf |
| cg15986030 | chr19 | COMP/TSS200/Island |
| cg02062744 | chr10 | ZFAND4,ZFAND4,ZFAND4,ZFAND4,ZFAND4/TSS1500,TSS1500,TSS1500,TSS1500,TSS1500/Island |
| cg14600016 | chr15 | -/-/- |
| cg20464360 | chr17 | HSF5/Body/Island |
| cg03611029 | chr3 | IQSEC1/TSS1500/S_Shore |
| cg13561879 | chr8 | UNC5D/TSS1500/Island |
| cg02188411 | chr1 | ATAD3B/Body/S_Shelf |
| cg22376913 | chr15 | IGF1R,IGF1R/Body,Body/- |
| cg12472449 | chr17 | BAIAP2,BAIAP2,BAIAP2,BAIAP2/Body,Body,Body,Body/N_Shelf |
| cg19052410 | chr15 | PWRN1/Body/- |
| cg23762517 | chr1 | HIVEP3,HIVEP3,HIVEP3,HIVEP3/1stExon,1stExon,5'UTR,5'UTR/S_Shore |
| cg03164627 | chr19 | KDM4B/Body/S_Shore |
| cg18486590 | chr14 | HSPA2,HSPA2/3'UTR,1stExon/S_Shore |
| cg06742719 | chr5 | PCDHGA2,PCDHGA3,PCDHGA1,PCDHGA3/Body,TSS200,Body,TSS200/N_Shore |
| cg00439658 | chr17 | GRIN2C/Body/Island |
| cg24633027 | chr5 | PCDHGA4,PCDHGA1,PCDHGA6,PCDHGA5,PCDHGB1,PCDHGA3,PCDHGA6,PCDHGA2,PCDHGB2,PCDHGB3/Body,Body,1stExon,Body,Body,Body,1stExon,Body,Body,Body/N_Shore |
| cg08743751 | chr5 | MAST4,MAST4/Body,Body/S_Shelf |
| cg04527880 | chr8 | PEBP4/Body/- |
| cg24056733 | chr5 | NA |
| cg19856897 | chr7 | -/-/S_Shore |
| cg07734149 | chr5 | SLC9A3,SLC9A3/Body,Body/S_Shelf |
| cg05527920 | chr4 | -/-/- |
| cg06096746 | chr20 | -/-/- |
| cg00714115 | chr19 | -/-/Island |
| cg20079992 | chr19 | SHANK1/Body/S_Shelf |
| cg06068362 | chr2 | LINC00486/Body/- |
| cg02281167 | chr6 | TRIM15/Body/Island |
| cg06893891 | chr4 | -/-/- |
| cg03716852 | chr19 | TRPM4/Body/Island |
| cg22736354 | chr6 | NHLRC1/1stExon/Island |
| cg01844642 | chr3 | GPR62/1stExon/Island |
| cg12868583 | chr22 | PNPLA3/Body/Island |
| cg09727935 | chr6 | LINC00577/TSS200/Island |
| cg11388320 | chr4 | PRDM8,PRDM8/5'UTR,5'UTR/Island |
| cg24450548 | chr21 | -/-/- |
| cg08885800 | chr1 | -/-/Island |
| cg09099868 | chr10 | -/-/N_Shore |
| cg15255403 | chr12 | -/-/S_Shore |
| cg20174472 | chr20 | SLCO4A1/5'UTR/- |
| cg26901661 | chr10 | -/-/- |
| cg14554415 | chr15 | MIR548H4,NOX5,NOX5,SPESP1,SPESP1/Body,5'UTR,1stExon,1stExon,5'UTR/Island |
| cg12480892 | chr5 | FBXL17/Body/- |
| cg22724998 | chr15 | -/-/N_Shore |
| cg06223834 | chr16 | ADCY9/Body/- |
| cg06654109 | chr10 | SLC18A3,CHAT,CHAT,CHAT/1stExon,TSS1500,TSS1500,5'UTR/Island |
| cg10477193 | chr10 | C10orf88/TSS1500/Island |
| cg00161791 | chr21 | -/-/N_Shore |
| cg00513288 | chr19 | -/-/N_Shore |
| cg19061171 | chr3 | FAM19A4,FAM19A4/TSS1500,TSS1500/Island |
| cg16604553 | chr9 | SLC1A1/TSS1500/N_Shore |
| cg06930511 | chr1 | CD1C/Body/- |
| cg09695851 | chr17 | -/-/Island |
| cg12234708 | chr7 | LHFPL3/Body/- |
| cg22858288 | chr11 | SLC22A18,SLC22A18/Body,Body/Island |
| cg25283626 | chr12 | -/-/Island |
| cg07772516 | chr15 | TMOD2,TMOD2/3'UTR,3'UTR/- |
| cg00587228 | chr19 | HCN2/Body/Island |
| cg19991948 | chr10 | TIAL1,TIAL1/3'UTR,3'UTR/- |
| cg10249828 | chr1 | LHX8,LHX8/1stExon,5'UTR/N_Shore |
| cg15747595 | chr8 | TSPYL5/1stExon/Island |
| cg20119148 | chr19 | PDE4C/5'UTR/Island |
| cg06674056 | chr11 | NRXN2,NRXN2/Body,Body/S_Shore |
| cg14348757 | chr6 | OPRM1,OPRM1,OPRM1,OPRM1,OPRM1,OPRM1,OPRM1,OPRM1,OPRM1,OPRM1,OPRM1,OPRM1,OPRM1,OPRM1,OPRM1,OPRM1,OPRM1,OPRM1,OPRM1,OPRM1,OPRM1,OPRM1,OPRM1,OPRM1,OPRM1,OPRM1,OPRM1,OPRM1,OPRM1,OPRM1/5'UTR,5'UTR,5'UTR,5'UTR,1stExon,1stExon,1stExon,1stExon,5'UTR,5'UTR,5'UTR,5'UTR,5'UTR,5'UTR,5'UTR,1stExon,1stExon,1stExon,1stExon,1stExon,1stExon,1stExon,5'UTR,Body,Body,Body,Body,Body,Body,Body/Island |
| cg22277749 | chr10 | VSTM4/Body/- |
| cg14318467 | chr10 | SLIT1/Body/- |
| cg17243289 | chr18 | SMAD2,SMAD2,SMAD2/TSS1500,TSS1500,TSS1500/Island |
| cg20066118 | chr2 | DCDC2C/1stExon/Island |
| cg09232937 | chr5 | IRX1/TSS200/Island |
| cg11630226 | chr8 | LY6K,LY6K,LY6K,LY6K,LY6K,LY6K/1stExon,1stExon,5'UTR,1stExon,5'UTR,5'UTR/Island |
| cg17955564 | chr5 | RANBP17/TSS1500/N_Shore |
| cg06097079 | chr2 | CHRND,CHRND,CHRND,CHRND/Body,Body,Body,Body/- |
| cg15219543 | chr7 | -/-/- |
| cg03994651 | chr19 | LMTK3/Body/Island |
| cg08411860 | chr3 | VWA5B2/Body/Island |
| cg17861230 | chr19 | PDE4C/Body/Island |
| cg18881380 | chr2 | IL1R1,IL1R1/TSS1500,5'UTR/N_Shore |
| cg18933331 | chr1 | -/-/S_Shore |
| cg08005858 | chr1 | CACNA1E,CACNA1E,CACNA1E/Body,Body,Body/- |
| cg23369111 | chr18 | C18orf26/TSS200/- |
| cg27206603 | chr16 | SHISA9/Body/- |
| cg01289480 | chr15 | KLF13,KLF13/Body,Body/- |
| cg27352764 | chr21 | SETD4,SETD4,SETD4,SETD4,SETD4/Body,Body,Body,Body,Body/- |
| cg18459154 | chr20 | -/-/Island |
| cg07017875 | chr5 | PCDHGA4,PCDHGA9,PCDHGA1,PCDHGB1,PCDHGB6,PCDHGB6,PCDHGB3,PCDHGA6,PCDHGA8,PCDHGA5,PCDHGB4,PCDHGA3,PCDHGA2,PCDHGB2,PCDHGA7,PCDHGB5/Body,Body,Body,Body,1stExon,1stExon,Body,Body,Body,Body,Body,Body,Body,Body,Body,Body/Island |
| cg26750073 | chr5 | -/-/- |
| cg24490801 | chr8 | -/-/- |
| cg12959488 | chr16 | -/-/S_Shore |
| cg00667789 | chr1 | TACSTD2/1stExon/Island |
| cg02715006 | chr5 | PCDHGA4,PCDHGA11,PCDHGA9,PCDHGA1,PCDHGB1,PCDHGB6,PCDHGB3,PCDHGA11,PCDHGB7,PCDHGA6,PCDHGA8,PCDHGA11,PCDHGA10,PCDHGA5,PCDHGB4,PCDHGA3,PCDHGA2,PCDHGB2,PCDHGA7,PCDHGB5/Body,Body,Body,Body,Body,Body,Body,1stExon,Body,Body,Body,1stExon,Body,Body,Body,Body,Body,Body,Body,Body/S_Shore |
| cg10235817 | chr4 | ADRA2C/1stExon/Island |
| cg04007813 | chr13 | LMO7,LMO7,LMO7/5'UTR,Body,Body/- |
| cg02755938 | chr18 | CHMP1B,GNAL,GNAL,GNAL/TSS1500,Body,Body,Body/N_Shore |
| cg05064829 | chr1 | -/-/- |
| cg01314359 | chr15 | PWAR1/Body/- |
| cg26305174 | chr7 | SLC12A9,TRIP6/Body,TSS1500/N_Shore |
| cg22070880 | chr22 | -/-/Island |
| cg18393958 | chr11 | FAM181B,FAM181B/1stExon,3'UTR/N_Shore |
| cg22087053 | chr5 | PCDHGA4,PCDHGB3,PCDHGA1,PCDHGA5,PCDHGB1,PCDHGA3,PCDHGB3,PCDHGA2,PCDHGB2/Body,1stExon,Body,Body,Body,Body,1stExon,Body,Body/Island |
| cg07573085 | chr4 | LETM1/Body/S_Shelf |
| cg16330755 | chr10 | C10orf32,C10orf32/TSS200,TSS200/N_Shore |
| cg24077102 | chr11 | C11orf85,C11orf85/TSS200,TSS200/Island |
| cg01634328 | chr4 | -/-/- |
| cg21237828 | chr16 | CNGB1,CNGB1/Body,Body/- |
| cg20673321 | chr19 | ZNF541/Body/S_Shore |
| cg05553829 | chr8 | SDR16C6/1stExon/- |
| cg06549686 | chr12 | LOC100996671,LOC100996671,LOC100996671/Body,Body,Body/- |
| cg13218710 | chr5 | SHROOM1/Body/Island |
| cg19407095 | chr13 | SOX1/TSS200/Island |
| cg01327147 | chr9 | KIAA1161/Body/Island |
| cg19349362 | chr7 | CEP41,CEP41,CEP41,CEP41,CEP41/Body,Body,Body,Body,Body/- |
| cg21921904 | chr7 | CYTH3/Body/- |
| cg01205295 | chr16 | JPH3,JPH3/Body,Body/- |
| cg08209133 | chr4 | SLC10A4/1stExon/Island |
| cg01418527 | chr7 | FSCN1/TSS1500/N_Shore |
| cg09596405 | chr1 | C1orf94,C1orf94,C1orf94/5'UTR,1stExon,5'UTR/S_Shore |
| cg17481903 | chr5 | -/-/N_Shore |
| cg03861347 | chr20 | SULF2,SULF2,SULF2/5'UTR,5'UTR,5'UTR/N_Shore |
| cg15656834 | chr11 | -/-/Island |
| cg14820504 | chr3 | -/-/- |
| cg15225071 | chr11 | BARX2/Body/S_Shore |
| cg23081781 | chr7 | GCC1/TSS1500/Island |
| cg23612932 | chr19 | -/-/- |
| cg01887148 | chr7 | IKZF1,IKZF1,IKZF1,IKZF1,IKZF1,IKZF1,IKZF1,IKZF1,IKZF1,IKZF1,IKZF1,IKZF1,IKZF1,IKZF1,IKZF1,IKZF1,IKZF1/Body,Body,Body,Body,Body,Body,Body,Body,Body,Body,Body,Body,Body,Body,Body,Body,Body/- |
| cg03641225 | chr1 | DIRAS3/Body/N_Shore |
| cg15384365 | chr1 | SOX13/5'UTR/- |
| cg14794655 | chr2 | C2orf73/Body/S_Shore |
| cg01768131 | chr17 | SIRT7,PCYT2/3'UTR,TSS1500/S_Shore |
| cg04417860 | chr6 | -/-/- |
| cg13514175 | chr17 | SERPINF1/Body/- |
| cg21149269 | chr1 | -/-/- |
| cg27236331 | chr15 | SPESP1,NOX5,SPESP1,NOX5,NOX5,NOX5,MIR548H4/5'UTR,5'UTR,1stExon,1stExon,Body,Body,Body/Island |
| cg24698979 | chr17 | ARHGAP23/Body/Island |
| cg11600454 | chr7 | THAP5,THAP5,THAP5,THAP5,THAP5,DNAJB9,THAP5,THAP5/Body,5'UTR,5'UTR,5'UTR,5'UTR,TSS1500,1stExon,1stExon/N_Shore |
| cg18071865 | chr7 | EGFR,EGFR,EGFR/Body,Body,Body/- |
| cg13858863 | chr19 | VN1R4/TSS1500/- |
| cg20294304 | chr6 | HMGA1,HMGA1,HMGA1,HMGA1,HMGA1/TSS1500,TSS1500,TSS1500,TSS1500,TSS1500/Island |
| cg01171887 | chr14 | -/-/N_Shore |
| cg18926249 | chr4 | -/-/- |
| cg05660634 | chr15 | -/-/- |
| cg22906442 | chr1 | NADK,NADK,NADK,NADK/TSS200,Body,Body,Body/N_Shore |
| cg22645278 | chr19 | ZNF560/TSS1500/S_Shore |
| cg03614034 | chr1 | -/-/- |
| cg23527621 | chr3 | ECE2,CAMK2N2/Body,3'UTR/Island |
| cg00807197 | chr12 | TMEM132C/Body/- |
| cg03776853 | chr22 | -/-/N_Shore |
| cg10189850 | chr5 | -/-/- |
| cg17758563 | chr6 | ATXN1,ATXN1/Body,Body/N_Shelf |
| cg22108374 | chr15 | CCDC33/Body/- |
| cg20515136 | chr3 | IL12A/Body/S_Shore |
| cg14611683 | chr1 | EIF2B3,EIF2B3/TSS1500,TSS1500/S_Shore |
| cg00075507 | chr17 | SARM1/Body/Island |
| cg02299710 | chr6 | RAET1G,RAET1G/TSS1500,TSS1500/N_Shore |
| cg16924010 | chr3 | -/-/S_Shelf |
| cg13307649 | chr14 | -/-/- |
| cg07262635 | chr10 | CACNB2,CACNB2,CACNB2,CACNB2,CACNB2,CACNB2,CACNB2,CACNB2,CACNB2/Body,Body,Body,Body,Body,TSS200,Body,Body,Body/- |
| cg01049205 | chr10 | LRIT1/Body/Island |
| cg20272935 | chr11 | UNC93B1/Body/S_Shore |
| cg26685941 | chr13 | ABCC4,ABCC4/Body,Body/N_Shore |
| cg22920501 | chr2 | FAM59B/Body/N_Shore |
| cg09461530 | chr19 | -/-/S_Shelf |
| cg08161480 | chr1 | -/-/S_Shelf |
| cg18546989 | chr6 | ARID1B,ARID1B/Body,Body/- |
| cg10036013 | chr7 | FOXK1/Body/- |
| cg04208403 | chr16 | ZNF423/Body/N_Shore |
| cg01302656 | chr14 | C14orf23/Body/Island |
| cg02419123 | chr8 | -/-/- |
| cg14692377 | chr17 | SLC6A4,SLC6A4/1stExon,5'UTR/Island |
| cg15949925 | chr19 | HSPBP1,HSPBP1,HSPBP1/Body,Body,Body/- |
| cg01162877 | chr1 | RERE,RERE/Body,Body/- |
| cg02204688 | chr19 | TTYH1,TTYH1,TTYH1/TSS1500,TSS1500,TSS1500/N_Shore |
| cg06675893 | chr18 | CELF4,CELF4,CELF4,CELF4/Body,Body,Body,Body/- |
| cg11659357 | chr7 | EGFR,EGFR,EGFR,EGFR/Body,Body,Body,Body/- |
| cg04655320 | chr12 | -/-/- |
| cg14373322 | chr19 | STRN4,STRN4,STRN4,STRN4/ExonBnd,ExonBnd,Body,Body/- |
| cg25616918 | chr2 | -/-/- |
| cg23414001 | chr20 | TRPC4AP,TRPC4AP/TSS1500,TSS1500/S_Shore |
| cg16968057 | chr6 | SOBP/Body/Island |
| cg08380618 | chr14 | -/-/- |
| cg11209121 | chr2 | NR4A2,NR4A2/Body,ExonBnd/S_Shore |
| cg22253012 | chr17 | -/-/- |
| cg06533577 | chr7 | -/-/- |
| cg24368226 | chr15 | ADAMTS17/Body/- |
| cg24436365 | chr6 | -/-/- |
| cg03308652 | chr3 | -/-/Island |
| cg12968684 | chr5 | EBF1,EBF1,EBF1/Body,Body,Body/- |
| cg15585987 | chr8 | SNTG1,SNTG1/5'UTR,1stExon/S_Shore |
| cg27515922 | chr16 | -/-/- |
| cg09935512 | chr2 | TEKT4,TEKT4,LOC442028/TSS1500,TSS1500,Body/N_Shore |
| cg08424749 | chr6 | PRRT1/Body/N_Shore |
| cg27076669 | chr12 | CPM,CPM,CPM/TSS1500,TSS1500,5'UTR/Island |
| cg25654705 | chr4 | -/-/N_Shelf |
| cg12610917 | chr19 | IRF2BP1/1stExon/Island |
| cg23268197 | chr19 | INSL3/1stExon/Island |
| cg21916100 | chr11 | APIP,PDHX,PDHX,PDHX/TSS1500,Body,Body,Body/S_Shore |
| cg02663839 | chr16 | -/-/- |
| cg24415728 | chr8 | -/-/S_Shore |
| cg06737494 | chr3 | GHSR,GHSR/TSS1500,TSS1500/Island |
| cg03169527 | chr3 | C3orf31/TSS1500/S_Shore |
| cg18758922 | chr22 | CACNG2/TSS1500/S_Shore |
| cg02893550 | chr16 | -/-/Island |
| cg15194935 | chr19 | ICAM5/Body/Island |
| cg23766254 | chr17 | FAM171A2/Body/Island |
| cg11210069 | chr5 | PCDHGA4,PCDHGA6,PCDHGA1,PCDHGA5,PCDHGB1,PCDHGA3,PCDHGA2,PCDHGB2,PCDHGA7,PCDHGA7,PCDHGB3/Body,Body,Body,Body,Body,Body,Body,Body,TSS1500,TSS1500,Body/N_Shore |
| cg21620282 | chr14 | CHGA,CHGA,CHGA,CHGA/5'UTR,5'UTR,1stExon,1stExon/Island |
| cg11551901 | chr10 | SEC31B/Body/- |
| cg11399640 | chr15 | -/-/- |
| cg27535616 | chr15 | -/-/- |
| cg24380977 | chr19 | -/-/- |
| cg14943847 | chr21 | RCAN1,RCAN1,RCAN1,RCAN1,RCAN1,RCAN1/TSS1500,TSS1500,Body,Body,Body,5'UTR/- |
| cg14570348 | chr19 | MIR1268A/Body/N_Shelf |
| cg12802086 | chr1 | MECR,MECR/TSS1500,TSS1500/S_Shore |
| cg23061027 | chr6 | PRRT1/3'UTR/N_Shore |
| cg09616647 | chr3 | VWA5B2,MIR1224/Body,TSS200/Island |
| cg01199494 | chr20 | EMILIN3/Body/N_Shore |
| cg17588585 | chr7 | -/-/N_Shore |
| cg04886950 | chr10 | PRKG1,PRKG1/Body,Body/- |
| cg14077898 | chr12 | SLC17A8,SLC17A8/TSS200,TSS200/- |
| cg27302051 | chr12 | -/-/- |
| cg04807108 | chr15 | -/-/- |
| cg08864344 | chr1 | -/-/Island |
| cg21763814 | chr6 | -/-/- |
| cg05275277 | chr12 | RIMBP2/5'UTR/- |
| cg17009978 | chr3 | VWA5B2,MIR1224/Body,TSS200/N_Shore |
| cg10243030 | chr20 | -/-/- |
| cg04479264 | chr19 | -/-/- |
| cg14359787 | chr19 | ANGPTL4,ANGPTL4,ANGPTL4/TSS1500,TSS1500,TSS1500/N_Shore |
| cg18006975 | chr5 | LOC100505841/Body/- |
| cg16276053 | chr12 | TMTC1,TMTC1/Body,Body/- |
| cg14674720 | chr2 | -/-/Island |
| cg06470855 | chr13 | -/-/Island |
| cg21995847 | chr1 | PSMD4/Body/- |
| cg03947688 | chr5 | ANKRD34B/TSS200/Island |
| cg25889998 | chr17 | -/-/- |
| cg04931708 | chr7 | FEZF1,FEZF1,FEZF1-AS1/TSS200,TSS200,Body/S_Shore |
| cg11842367 | chr1 | MGC27382/Body/- |
| cg25756406 | chr10 | -/-/Island |
| cg10192587 | chr5 | -/-/- |
| cg04803208 | chr8 | LOC100130298/Body/- |
| cg21771140 | chr8 | -/-/- |
| cg15557036 | chr17 | -/-/Island |
| cg10703784 | chr12 | -/-/N_Shore |
| cg01754082 | chr20 | PCSK2,PCSK2,PCSK2/TSS1500,TSS1500,TSS200/Island |
| cg01895805 | chr8 | TRAPPC9,TRAPPC9/Body,Body/- |
| cg16219603 | chr8 | PENK/TSS1500/Island |
| cg26387435 | chr7 | CNTNAP2/Body/- |
| cg15507683 | chr15 | TCF12,TCF12,TCF12,TCF12,TCF12,TCF12,TCF12/Body,Body,Body,Body,Body,Body,Body/- |
| cg07160033 | chr1 | -/-/- |
| cg26333393 | chr10 | CPXM2/TSS1500/S_Shore |
| cg10644701 | chr12 | -/-/- |
| cg26375473 | chr5 | PCDHGA4,PCDHGA11,PCDHGA11,PCDHGA9,PCDHGA1,PCDHGB1,PCDHGB6,PCDHGA12,PCDHGB3,PCDHGB7,PCDHGA6,PCDHGA8,PCDHGA10,PCDHGA12,PCDHGA5,PCDHGB4,PCDHGA3,PCDHGA2,PCDHGA7,PCDHGB2,PCDHGB5/Body,Body,Body,Body,Body,Body,Body,1stExon,Body,Body,Body,Body,Body,1stExon,Body,Body,Body,Body,Body,Body,Body/Island |
| cg21690805 | chr22 | TRABD/5'UTR/N_Shore |
| cg01296603 | chr1 | LRP8,LRP8,LRP8,LRP8/Body,Body,Body,Body/- |
| cg09066349 | chr20 | -/-/- |
| cg19392551 | chr10 | AKR1E2/TSS200/Island |
| cg19419575 | chr12 | SP1,SP1,SP1/3'UTR,3'UTR,3'UTR/- |
| cg11471138 | chr5 | -/-/N_Shelf |
| cg07715257 | chr1 | EPHA2/Body/N_Shelf |
| cg15389227 | chr7 | CADPS2,CADPS2,CADPS2/Body,Body,Body/- |
| cg18158855 | chr20 | SLC13A3,SLC13A3,SLC13A3,SLC13A3,SLC13A3/Body,Body,Body,Body,Body/- |
| cg13972648 | chr2 | -/-/- |
| cg22679740 | chr3 | -/-/S_Shelf |
| cg04028695 | chr10 | -/-/- |
| cg03653511 | chr5 | B4GALT7/TSS1500/N_Shore |
| cg14811319 | chr6 | SPACA1/TSS200/Island |
| cg13654588 | chr10 | PRLHR/Body/Island |
| cg03235758 | chr17 | MYCBPAP/Body/- |
| cg09017434 | chr5 | MARCH11/1stExon/Island |
| cg15966954 | chr5 | IL9/TSS200/- |
| cg11220950 | chr16 | SYNGR3/Body/Island |
| cg14854355 | chr17 | SARM1/Body/Island |
| cg10784813 | chr16 | SOCS1/3'UTR/Island |
| cg12781086 | chr7 | BMPER/Body/Island |
| cg18813588 | chr5 | MCC,MCC/Body,Body/- |
| cg24093698 | chr11 | NTM/Body/- |
| cg01013171 | chr14 | -/-/- |
| cg16005271 | chr10 | -/-/N_Shore |
| cg10361013 | chr10 | RASGEF1A/TSS1500/- |
| cg07490692 | chr21 | -/-/- |
| cg21093945 | chr1 | -/-/- |
| cg06896909 | chr7 | TMEM196/TSS1500/S_Shore |
| cg17152981 | chr6 | GPR6/TSS1500/Island |
| cg02911246 | chr17 | -/-/- |
| cg03054277 | chr1 | OBSCN,OBSCN/Body,Body/Island |
| cg17923553 | chr4 | SOD3/TSS200/- |
| cg04455670 | chr4 | PALLD,PALLD,PALLD,PALLD/Body,Body,Body,Body/S_Shelf |
| cg02752915 | chr17 | DOC2B/1stExon/Island |
| cg25418748 | chr5 | RUFY1/TSS1500/N_Shore |
| cg15504461 | chr3 | -/-/N_Shore |
| cg09333820 | chr3 | CD200,CD200/TSS1500,TSS1500/N_Shore |
| cg19646200 | chr12 | GALNT9/Body/S_Shore |
| cg07884764 | chr11 | CCDC88B/TSS200/N_Shelf |
| cg15626285 | chr12 | C1S,C1S/TSS200,TSS200/- |
| cg23995914 | chr4 | ZNF518B/TSS200/Island |
| cg14913178 | chr6 | -/-/- |
| cg06942685 | chr19 | ZNF542,ZNF542,ZNF542,ZNF542/TSS1500,TSS1500,TSS200,TSS200/Island |
| cg25414165 | chr10 | C10orf11/TSS200/- |
| cg25713558 | chr14 | DIO2,DIO2,DIO2,DIO2,DIO2,DIO2-AS1/TSS200,TSS1500,TSS1500,TSS1500,5'UTR,Body/- |
| cg04858539 | chr22 | LARGE,LARGE/Body,Body/- |
| cg24871743 | chr1 | DIRAS3/Body/Island |
| cg00417151 | chr20 | RRBP1,RRBP1/Body,Body/S_Shelf |
| cg16083551 | chr16 | -/-/Island |
| cg22201540 | chr9 | -/-/- |
| cg13723336 | chr8 | -/-/- |
| cg19320261 | chr1 | ROR1,ROR1/Body,Body/- |
| cg11622231 | chr6 | SOBP/Body/Island |
| cg01287975 | chr7 | TAC1,TAC1,TAC1,TAC1/TSS200,TSS200,TSS200,TSS200/Island |
| cg19671120 | chr2 | CNGA3,CNGA3,CNGA3,CNGA3/1stExon,1stExon,5'UTR,5'UTR/Island |
| cg11290378 | chr10 | CCDC172/5'UTR/- |
| cg25983380 | chr20 | GNAS,GNAS,GNAS,GNAS,GNAS,GNAS,GNAS,GNAS/3'UTR,TSS1500,TSS1500,Body,TSS1500,3'UTR,TSS1500,Body/Island |
| cg27631817 | chr6 | -/-/- |
| cg00254679 | chr6 | MAP3K5/Body/- |
| cg09530779 | chr3 | BTLA,BTLA,BTLA,BTLA/1stExon,1stExon,5'UTR,5'UTR/- |
| cg21154795 | chr12 | -/-/- |
| cg07408456 | chr19 | PGLYRP2/TSS1500/- |
| cg27570738 | chr12 | -/-/Island |
| cg20505565 | chr2 | -/-/- |
| cg26277237 | chr9 | KANK1,KANK1,KANK1/5'UTR,5'UTR,5'UTR/- |
| cg02983424 | chr15 | -/-/- |
| cg08005692 | chr1 | ASH1L/5'UTR/- |
| cg05702347 | chr6 | -/-/- |
| cg09639931 | chr17 | ZPBP2,ZPBP2/TSS200,TSS200/Island |
| cg07766263 | chr3 | SLC7A14/5'UTR/Island |
| cg09788125 | chr15 | -/-/- |
| cg11918171 | chr3 | ZBED2,CD96,CD96/TSS1500,Body,Body/- |
| cg05404236 | chr13 | IRS2/1stExon/Island |
| cg26140120 | chr8 | FAM83A,FAM83A/Body,Body/Island |
| cg03975235 | chr22 | GALR3/Body/Island |
| cg14968926 | chr5 | CWC27,CWC27/Body,Body/- |
| cg12887220 | chr1 | BPNT1,BPNT1,BPNT1,BPNT1,RNU5F-1/TSS1500,TSS1500,TSS1500,TSS1500,Body/N_Shelf |
| cg21520111 | chr19 | TRPM4/Body/Island |
| cg13673455 | chr5 | PCSK1,PCSK1,LOC101929710/Body,Body,Body/- |
| cg21186757 | chr2 | -/-/- |
| cg11432303 | chr5 | HTR1A/TSS1500/S_Shore |
| cg03428193 | chr12 | B4GALNT3/Body/- |
| cg07892422 | chr12 | HOXC13/1stExon/Island |
| cg14043816 | chr1 | -/-/- |
| cg25745729 | chr10 | -/-/- |
| cg26449130 | chr4 | -/-/- |
| cg21822173 | chr2 | AMMECR1L,AMMECR1L/5'UTR,5'UTR/- |
| cg23900763 | chr11 | PSMD13,PSMD13/Body,Body/- |
| cg04584103 | chr14 | ASB2/Body/Island |
| cg21931419 | chr14 | TMEM90A/TSS1500/Island |
| cg12693179 | chr12 | LGR5,LGR5,LGR5,LGR5/Body,Body,Body,Body/- |
| cg02134705 | chr3 | WWTR1/5'UTR/- |
| cg24084872 | chr3 | ZBTB20-AS3,ZBTB20,ZBTB20,ZBTB20/TSS1500,Body,5'UTR,5'UTR/- |
| cg20751926 | chr16 | MMP15/Body/Island |
| cg15851014 | chr4 | TLR3/TSS1500/- |
| cg19540560 | chr8 | SGCZ/TSS1500/S_Shore |
| cg00688297 | chr8 | LRRC24,MGC70857/5'UTR,3'UTR/Island |
| cg05694021 | chr12 | -/-/- |
| cg15378061 | chr7 | -/-/- |
| cg02329226 | chr11 | PACS1/Body/- |
| cg24853724 | chr7 | TRIL/1stExon/Island |
| cg27565473 | chr3 | -/-/Island |
| cg06758240 | chr11 | -/-/- |
| cg24540066 | chr1 | -/-/- |
| cg08230167 | chr9 | LURAP1L-AS1,LURAP1L/TSS200,Body/- |
| cg10960709 | chr7 | RAB19/Body/- |
| cg08762472 | chr5 | -/-/- |
| cg22800233 | chr10 | -/-/N_Shore |
| cg10097464 | chr15 | ANKDD1A/Body/Island |
| cg01746241 | chr9 | KIAA1161/Body/Island |
| cg00603340 | chr5 | TRIM7,TRIM7,TRIM7,TRIM7,TRIM7/3'UTR,3'UTR,3'UTR,3'UTR,3'UTR/N_Shore |
| cg17299258 | chr19 | HMHA1,HMHA1,HMHA1,HMHA1/TSS1500,Body,Body,Body/S_Shore |
| cg02024925 | chr13 | -/-/S_Shelf |
| cg01196788 | chr2 | -/-/N_Shore |
| cg25472283 | chr2 | ASAP2,ASAP2/Body,Body/- |
| cg07806886 | chr3 | STXBP5L/TSS200/Island |
| cg11201710 | chr3 | FGF12/Body/Island |
| cg19702785 | chr20 | KCNS1/Body/Island |
| cg00153289 | chr8 | -/-/- |
| cg07082267 | chr16 | -/-/- |
| cg00667751 | chr6 | -/-/Island |
| cg16533838 | chr11 | -/-/N_Shelf |
| cg05457768 | chr6 | -/-/N_Shelf |
| cg14862806 | chr17 | -/-/Island |
| cg27315249 | chr16 | NA |
| cg18608703 | chr7 | CFTR/TSS1500/- |
| cg22650458 | chr7 | C7orf41/Body/- |
| cg01027976 | chr22 | -/-/Island |
| cg09113853 | chr4 | LINC01091/Body/- |
| cg11953609 | chr1 | -/-/N_Shore |
| cg18920097 | chr15 | GJD2/Body/N_Shore |
| cg07132557 | chr8 | TPD52,TPD52,TPD52,TPD52,TPD52,TPD52,TPD52,TPD52,TPD52,TPD52,TPD52,TPD52/Body,Body,Body,Body,Body,Body,Body,Body,Body,Body,Body,Body/- |
| cg07341306 | chr8 | -/-/- |
| cg12259537 | chr19 | ZNF606,ZNF606/5'UTR,1stExon/S_Shore |
| cg05157335 | chr2 | -/-/- |
| cg13872789 | chr5 | SLC36A1,SLC36A1,SLC36A1/Body,Body,Body/- |
| cg10475970 | chr5 | PCDHGA4,PCDHGA6,PCDHGA1,PCDHGA5,PCDHGB1,PCDHGB4,PCDHGA3,PCDHGA2,PCDHGA8,PCDHGA7,PCDHGB2,PCDHGA8,PCDHGB3/Body,Body,Body,Body,Body,Body,Body,Body,1stExon,Body,Body,1stExon,Body/Island |
| cg01301252 | chr5 | PCDHGA2,PCDHGA3,PCDHGA3,PCDHGA1/Body,1stExon,1stExon,Body/N_Shore |
| cg12081325 | chr1 | EIF2B3,EIF2B3/Body,Body/- |
| cg26528357 | chr14 | -/-/- |
| cg10107890 | chr2 | -/-/Island |
| cg19190593 | chr6 | LY86,LOC285780/TSS200,Body/- |
| cg17903450 | chr6 | SCGN/TSS200/N_Shore |
| cg09726198 | chr3 | -/-/- |
| cg11747251 | chr11 | CREBZF,CREBZF,CREBZF,CREBZF,CREBZF,CREBZF/3'UTR,1stExon,Body,Body,Body,Body/N_Shore |
| cg18236477 | chr13 | ATP8A2/Body/Island |
| cg21218758 | chr19 | ELAVL3,ELAVL3/TSS200,TSS200/Island |
| cg05371578 | chr3 | ZIC1/1stExon/Island |
| cg17303833 | chr10 | ZNF518A/TSS200/N_Shore |
| cg16544169 | chr8 | NKX2-6/TSS1500/Island |
| cg03607117 | chr3 | SFMBT1,SFMBT1,SFMBT1/TSS1500,TSS1500,TSS1500/Island |
| cg25045526 | chr7 | FEZF1,FEZF1,FEZF1,FEZF1,FEZF1-AS1/1stExon,1stExon,5'UTR,5'UTR,Body/Island |
| cg12765028 | chr4 | -/-/Island |
| cg14811105 | chr2 | NR4A2/Body/Island |
| cg13848598 | chr10 | ADRB1/1stExon/Island |
| cg00841760 | chr7 | PTPRN2,PTPRN2,PTPRN2/Body,Body,Body/Island |
| cg16456870 | chr1 | PER3,PER3,PER3,PER3,PER3/TSS200,TSS200,TSS200,TSS200,TSS200/Island |
| cg23680086 | chr5 | PCDHGA8,PCDHGB5,PCDHGA9,PCDHGB6,PCDHGB6,PCDHGA1,PCDHGA2,PCDHGA3,PCDHGB1,PCDHGA4,PCDHGB2,PCDHGA5,PCDHGB3,PCDHGA6,PCDHGA7,PCDHGB4/Body,Body,Body,TSS200,TSS200,Body,Body,Body,Body,Body,Body,Body,Body,Body,Body,Body/Island |
| cg20742389 | chr19 | GIPC1,GIPC1,GIPC1,GIPC1,GIPC1,GIPC1/Body,Body,Body,Body,Body,Body/Island |
| cg03126058 | chr1 | PRDM16,PRDM16/Body,Body/- |
| cg15619071 | chr14 | SLC25A21-AS1,SLC25A21,SLC25A21/Body,TSS1500,TSS1500/S_Shore |
| cg26220336 | chr20 | FAM83D/TSS1500/N_Shore |
| cg25694790 | chr20 | DSTN,DSTN/Body,Body/- |
| cg19146916 | chr9 | -/-/- |
| cg19501858 | chr2 | ST6GAL2,ST6GAL2,ST6GAL2/5'UTR,5'UTR,5'UTR/- |
| cg06962373 | chr13 | -/-/- |
| cg16306870 | chr3 | C3orf21/Body/- |
| cg27530239 | chr17 | CALCOCO2/5'UTR/S_Shore |
| cg05845403 | chr13 | DZIP1,DZIP1/Body,Body/Island |
| cg19599611 | chr15 | AQR/3'UTR/- |
| cg04986579 | chr19 | UNC13A/TSS1500/S_Shore |
| cg05412028 | chr13 | ABCC4,ABCC4/Body,Body/N_Shore |
| cg21429394 | chr12 | SLC17A8,SLC17A8,SLC17A8,SLC17A8/1stExon,5'UTR,1stExon,5'UTR/- |
| cg03028536 | chr12 | CUX2/Body/- |
| cg06333233 | chr2 | CREG2/3'UTR/- |
| cg03185704 | chr7 | LRRC61,ACTR3C,LRRC61/TSS200,5'UTR,TSS200/Island |
| cg14105637 | chr21 | KRTAP12-3,TSPEAR,TSPEAR/TSS200,Body,5'UTR/- |
| cg22398259 | chr8 | -/-/- |
| cg14087880 | chr10 | PPYR1/TSS1500/N_Shore |
| cg04436818 | chr10 | PAX2,PAX2,PAX2,PAX2,PAX2/3'UTR,3'UTR,3'UTR,3'UTR,3'UTR/N_Shore |
| cg00247870 | chr2 | -/-/- |
| cg21047367 | chr2 | GREB1/Body/Island |
| cg07254032 | chr5 | HCN1/1stExon/Island |
| cg03649834 | chr1 | SELP/Body/- |
| cg05548469 | chr4 | TRPC3,TRPC3/1stExon,Body/N_Shore |
| cg06691343 | chr8 | -/-/- |
| cg19392831 | chr10 | PRLHR/TSS1500/Island |
| cg14214717 | chr20 | -/-/- |
| cg05665581 | chr10 | FAM24A/TSS1500/- |
| cg10141715 | chr12 | SLC5A8/1stExon/Island |
| cg08443492 | chr21 | KRTAP10-6,TSPEAR,TSPEAR/TSS1500,Body,5'UTR/- |
| cg02228185 | chr17 | ASPA,ASPA/1stExon,Body/- |
| cg14162806 | chr22 | PDGFB/TSS1500/S_Shore |
| cg07758738 | chr18 | -/-/Island |
| cg27242132 | chr4 | PRDM8,PRDM8/5'UTR,5'UTR/Island |
| cg22013249 | chr2 | KLHL29/Body/- |
| cg21788281 | chr5 | -/-/Island |
| cg07502389 | chr8 | NEFM,NEFM/TSS200,TSS1500/Island |
| cg08878263 | chr16 | SLC9A5/Body/- |
| cg20558987 | chr20 | ARFGEF2/TSS1500/N_Shore |
| cg12004295 | chr15 | FAM189A1/Body/- |
| cg07719604 | chr16 | ELMO3,E2F4/TSS1500,3'UTR/N_Shore |
| cg15381304 | chr6 | GPR6/TSS200/Island |
| cg00151250 | chr8 | NECAB1/TSS1500/N_Shore |
| cg17111800 | chr12 | NINJ2,NINJ2,LOC100049716/Body,5'UTR,Body/- |
| cg20058308 | chr12 | -/-/- |
| cg08475411 | chr17 | -/-/- |
| cg15957394 | chr4 | AFAP1,AFAP1/TSS200,TSS200/Island |
| cg15726426 | chr1 | KCNA3/1stExon/Island |
| cg24061675 | chr12 | -/-/- |
| cg15936446 | chr5 | -/-/Island |
| cg00419702 | chr22 | BAIAP2L2/Body/Island |
| cg16281776 | chr1 | PRELP,PRELP/TSS1500,TSS1500/- |
| cg04807694 | chr2 | UBE2E3,UBE2E3,UBE2E3/5'UTR,5'UTR,1stExon/Island |
| cg00578039 | chr6 | C6orf25,C6orf25,C6orf25,C6orf25,C6orf25,C6orf25/Body,Body,Body,Body,Body,Body/N_Shore |
| cg09305680 | chr8 | UTP23/TSS1500/N_Shore |
| cg26773506 | chr11 | UBE4A,UBE4A/TSS1500,TSS1500/N_Shore |
| cg12094174 | chr6 | MICB/Body/Island |
| cg26888530 | chr4 | AFAP1,AFAP1/TSS200,TSS200/Island |
| cg15729315 | chr4 | APBB2,APBB2,APBB2/Body,Body,Body/- |
| cg09739017 | chr17 | CACNG1/1stExon/Island |
| cg08859675 | chr19 | PDE4A,PDE4A,PDE4A,PDE4A,PDE4A,PDE4A/5'UTR,1stExon,Body,Body,Body,Body/- |
| cg21750785 | chr6 | DCBLD1/Body/- |
| cg27239981 | chr6 | -/-/N_Shelf |
| cg04478095 | chr6 | -/-/Island |
| cg27366532 | chr16 | CREBBP,CREBBP/Body,Body/- |
| cg24592370 | chr12 | GALNT9,LOC100130238/Body,Body/N_Shore |
| cg17380795 | chr3 | -/-/- |
| cg01295203 | chr8 | PRDM14/TSS1500/Island |
| cg27320127 | chr2 | KCNK12/TSS1500/Island |
| cg01933040 | chr12 | TPH2/Body/- |
| cg05176199 | chr15 | -/-/- |
| cg00237547 | chr14 | KIAA0125/Body/- |
| cg27099280 | chr15 | CELF6,CELF6/1stExon,1stExon/Island |
| cg18633600 | chr12 | LRTM2,LRTM2,CACNA2D4,LRTM2/Body,Body,Body,Body/- |
| cg26975066 | chr11 | PGR,PGR,PGR,PGR,PGR,PGR,PGR,LOC101054525/TSS1500,TSS1500,TSS1500,TSS1500,TSS1500,TSS1500,TSS1500,Body/S_Shore |
| cg17679107 | chr8 | NSMCE2/Body/- |
| cg01770755 | chr15 | -/-/Island |
| cg09190051 | chr15 | ZNF280D,ZNF280D,ZNF280D/TSS1500,TSS1500,TSS1500/Island |
| cg23270757 | chr6 | OR2H1/5'UTR/- |
| cg13586791 | chr1 | TPM3,TPM3,TPM3,TPM3,TPM3,TPM3,TPM3,TPM3,TPM3,TPM3/TSS1500,Body,Body,Body,Body,Body,Body,Body,Body,Body/- |
| cg02415220 | chr19 | -/-/- |
| cg02305765 | chr12 | -/-/N_Shore |
| cg08638989 | chr11 | -/-/- |
| cg05519231 | chr1 | ECHDC2,ECHDC2,ECHDC2/TSS200,TSS200,TSS200/S_Shore |
| cg01236038 | chr14 | SLC8A3,SLC8A3,SLC8A3,SLC8A3,SLC8A3,SLC8A3,SLC8A3/5'UTR,Body,Body,Body,Body,Body,Body/- |
| cg25955180 | chr6 | PRRT1/3'UTR/N_Shore |
| cg15283904 | chr10 | PTCHD3/TSS200/Island |
| cg15480367 | chr14 | CHGA,CHGA/5'UTR,1stExon/Island |
| cg14575215 | chr12 | ANKLE2/Body/- |
| cg12757011 | chr2 | TBR1/3'UTR/S_Shore |
| cg12205270 | chr17 | CYTH1,CYTH1/Body,Body/- |
| cg09154591 | chr2 | VIT/TSS200/- |
| cg17158649 | chr13 | CLYBL-AS2,CLYBL,CLYBL/TSS1500,Body,Body/- |
| cg18200389 | chr6 | TRIM26/5'UTR/- |
| cg21787323 | chr11 | TMEM126B,DLG2/TSS1500,5'UTR/N_Shore |
| cg09959532 | chr3 | -/-/- |
| cg01066431 | chr10 | LOC642826/TSS1500/N_Shore |
| cg19761273 | chr17 | CSNK1D,CSNK1D/TSS1500,TSS1500/S_Shore |
| cg16884061 | chr12 | RAD52,RAD52,RAD52,RAD52,RAD52/Body,Body,Body,Body,Body/- |
| cg08157375 | chr1 | -/-/N_Shelf |
| cg22851420 | chr1 | HPCAL4/Body/Island |
| cg24501701 | chr12 | AVPR1A/1stExon/Island |
| cg08557795 | chr8 | CALB1/TSS200/- |
| cg26307725 | chr6 | -/-/- |
| cg20309061 | chr15 | CAPN3,CAPN3,CAPN3,CAPN3,CAPN3,CAPN3,CAPN3/Body,TSS1500,Body,Body,Body,Body,Body/- |
| cg18196063 | chr1 | GRHL3,GRHL3,GRHL3/Body,5'UTR,Body/S_Shelf |
| cg15365265 | chr1 | -/-/- |
| cg22809047 | chr2 | RPL31,RPL31,RPL31/TSS1500,TSS1500,TSS1500/Island |
| cg14900145 | chr20 | -/-/- |
| cg24214068 | chr8 | NEFM,NEFM/TSS1500,TSS200/Island |
| cg01565160 | chr20 | DIDO1/Body/Island |
| cg06647818 | chr22 | RFPL2/TSS1500/- |
| cg23601515 | chr17 | EVPL/Body/Island |
| cg24466241 | chr1 | ZYG11A/Body/Island |
| cg18221429 | chr7 | LRRC61,ACTR3C,LRRC61/TSS200,5'UTR,TSS200/Island |
| cg16087521 | chr7 | PDE1C/TSS1500/S_Shore |
| cg24444489 | chr15 | SNORD115-25,SNORD115-21,SNORD115-15/TSS200,Body,Body/- |
| cg24076884 | chr5 | PCDHAC2,PCDHA7,PCDHA12,PCDHA6,PCDHA10,PCDHA4,PCDHA11,PCDHA8,PCDHA6,PCDHA1,PCDHA2,PCDHA1,PCDHA9,PCDHA13,PCDHA5,PCDHAC1,PCDHA3,PCDHAC2,PCDHA10/1stExon,Body,Body,Body,Body,Body,Body,Body,Body,Body,Body,Body,Body,Body,Body,Body,Body,1stExon,Body/Island |
| cg01179137 | chr9 | -/-/- |
| cg15964874 | chr8 | -/-/- |
| cg06122864 | chr6 | MOG,MOG,MOG,MOG,MOG,MOG,MOG,MOG,MOG,MOG,MOG/Body,Body,Body,Body,Body,Body,Body,Body,Body,Body,Body/- |
| cg22902669 | chr17 | -/-/- |
| cg24608182 | chr1 | BOLA1/Body/Island |
| cg10320659 | chr20 | -/-/Island |
| cg23777302 | chr11 | -/-/Island |
| cg11945824 | chr6 | PRRT1/3'UTR/Island |
| cg19989581 | chr15 | -/-/- |
| cg09552588 | chr19 | -/-/- |
| cg12085698 | chr11 | SPON1/TSS200/N_Shore |
| cg21108174 | chr17 | -/-/- |
| cg07007312 | chr4 | DGKQ/Body/Island |
| cg09357926 | chr4 | F11-AS1/TSS200/S_Shore |
| cg16379910 | chr14 | -/-/- |
| cg21302951 | chr2 | KCNH7,KCNH7/TSS1500,TSS1500/- |
| cg06882058 | chr1 | SDCCAG8/Body/Island |
| cg23312086 | chr8 | -/-/- |
| cg10567964 | chr1 | KLHDC8A/Body/Island |
| cg09205236 | chr19 | -/-/- |
| cg02973171 | chr11 | FAM181B/1stExon/Island |
| cg03012854 | chr22 | RFPL1S,RFPL1/Body,TSS200/- |
| cg09279169 | chr19 | ZFP30/TSS1500/S_Shore |
| cg20024221 | chr19 | ZSCAN18,ZSCAN18/Body,Body/Island |
| cg10647509 | chr8 | ADRA1A,ADRA1A,ADRA1A,ADRA1A,ADRA1A,ADRA1A,ADRA1A,ADRA1A/5'UTR,5'UTR,5'UTR,5'UTR,1stExon,1stExon,1stExon,1stExon/Island |
| cg07159847 | chr12 | AGAP2,AGAP2/Body,Body/S_Shore |
| cg01495918 | chr2 | -/-/- |
| cg17632590 | chr1 | KIF26B/Body/- |
| cg03411341 | chr9 | CNTNAP3B,CNTNAP3P2/TSS1500,TSS1500/N_Shore |
| cg10500653 | chr1 | RHBDL2/TSS200/- |
| cg16541931 | chr10 | LOC100128811,GPR158/Body,TSS1500/Island |
| cg16012162 | chr1 | -/-/- |
| cg13075971 | chr17 | ITGB3/Body/S_Shelf |
| cg17018786 | chr15 | DISP2/Body/N_Shore |
| cg14524754 | chr17 | B3GNTL1/Body/N_Shelf |
| cg23752000 | chr17 | NPTX1/3'UTR/N_Shore |
| cg12764532 | chr13 | -/-/S_Shore |
| cg14885175 | chr5 | SLC23A1,SLC23A1/TSS1500,TSS1500/- |
| cg25273039 | chr7 | NXPH1/TSS200/Island |
| cg05722266 | chr2 | ITPRIPL1,ITPRIPL1,ITPRIPL1,ITPRIPL1/5'UTR,TSS200,TSS200,Body/S_Shore |
| cg07060551 | chr19 | SHANK1/Body/Island |
| cg18284157 | chr22 | CACNG2/TSS1500/S_Shore |
| cg24678117 | chr1 | -/-/S_Shore |
| cg21946496 | chr3 | PRR23A/1stExon/Island |
| cg14612428 | chr18 | GNAL,GNAL,GNAL,GNAL,GNAL/5'UTR,5'UTR,1stExon,1stExon,Body/Island |
| cg10816169 | chr11 | -/-/Island |
| cg04349084 | chr8 | -/-/- |
| cg00497679 | chr17 | -/-/N_Shelf |
| cg13793262 | chr10 | GPR26/Body/- |
| cg07105947 | chr1 | -/-/Island |
| cg00521883 | chr14 | -/-/- |
| cg09670562 | chr19 | MYH14,MYH14,MYH14/Body,Body,Body/- |
| cg21683459 | chr14 | NRXN3/Body/- |
| cg07923390 | chr6 | TCP11,TCP11/TSS200,TSS200/Island |
| cg05756933 | chr6 | GPR6/TSS200/Island |
| cg16477091 | chr17 | PPM1E/TSS1500/Island |
| cg03190737 | chr8 | -/-/- |
| cg17973328 | chr1 | -/-/- |
| cg20036244 | chr1 | RSBN1,RSBN1,AP4B1-AS1/TSS1500,TSS1500,Body/S_Shore |
| cg03969797 | chr15 | MKRN3,MKRN3/5'UTR,1stExon/- |
| cg05139788 | chr6 | TFAP2D/Body/N_Shore |
| cg17625893 | chr14 | -/-/S_Shore |
| cg23202262 | chr12 | -/-/N_Shore |
| cg07230471 | chr20 | JPH2/Body/S_Shelf |
| cg16750801 | chr10 | PTPRE/5'UTR/S_Shore |
| cg02741882 | chr16 | SEZ6L2,SEZ6L2,SEZ6L2,SEZ6L2/Body,Body,Body,Body/Island |
| cg05133340 | chr12 | GALNT9,GALNT9/Body,TSS1500/S_Shore |
| cg12484489 | chr19 | LOC100631378/TSS200/Island |
| cg06369624 | chr20 | KCNS1/Body/Island |
| cg06799152 | chr12 | LRP1/Body/- |
| cg00959431 | chr9 | -/-/Island |
| cg07934780 | chr7 | NXPH1/5'UTR/Island |
| cg27167868 | chr20 | -/-/- |
| cg23327200 | chr17 | TTYH2,TTYH2/5'UTR,Body/- |
| cg22794031 | chr10 | -/-/Island |
| cg06735326 | chr5 | MCTP1/Body/- |
| cg25942450 | chr5 | TLX3/TSS200/Island |
| cg07336350 | chr16 | -/-/Island |
| cg03071793 | chr16 | LITAF/TSS1500/S_Shore |
| cg14971895 | chr10 | GPR158,LOC100128811/Body,TSS200/Island |
| cg16717713 | chr14 | CCDC85C/1stExon/Island |
| cg01503065 | chr4 | DCHS2,DCHS2/1stExon,1stExon/Island |
| cg12104897 | chr22 | LINC01310/Body/- |
| cg01158574 | chr2 | NTSR2/1stExon/Island |
| cg10390211 | chr13 | LINC00282,LINC00282/TSS1500,TSS1500/- |
| cg06458132 | chr9 | PTPN3,PTPN3,PTPN3,PTPN3/TSS1500,TSS1500,Body,Body/- |
| cg27639942 | chr11 | -/-/Island |
| cg04522339 | chr9 | MIR4540,PAX5,PAX5,PAX5,PAX5,PAX5,PAX5,PAX5,PAX5,PAX5,PAX5,PAX5,PAX5,PAX5/TSS1500,Body,Body,Body,Body,Body,Body,Body,Body,Body,Body,Body,Body,Body/- |
| cg16589260 | chr7 | EGFR,EGFR,EGFR/Body,Body,Body/- |
| cg26890189 | chr19 | SLC8A2/Body/Island |
| cg16771104 | chr3 | HESX1,HESX1/1stExon,5'UTR/- |
| cg23832334 | chr3 | C3orf56/TSS200/- |
| cg06024696 | chr10 | ZNF365,ZNF365/Body,5'UTR/- |
| cg12586992 | chr11 | -/-/- |
| cg04310491 | chr18 | CELF4,CELF4,CELF4,CELF4/Body,Body,Body,Body/S_Shelf |
| cg24489074 | chr10 | -/-/- |
| cg01368488 | chr10 | -/-/Island |
| cg16345647 | chr21 | DSCR6/TSS1500/Island |
| cg10189695 | chr4 | GPR78,GPR78/1stExon,5'UTR/Island |
| cg08534628 | chr13 | -/-/Island |
| cg20022869 | chr20 | NPEPL1/TSS1500/N_Shore |
| cg05535335 | chr2 | -/-/- |
| cg00529943 | chr21 | -/-/S_Shore |
| cg07462745 | chr6 | -/-/N_Shore |
| cg23347958 | chr17 | DHX8/TSS200/Island |
| cg14120436 | chr15 | GNB5/5'UTR/- |
| cg02855309 | chr16 | -/-/Island |
| cg21090923 | chr16 | HBQ1/TSS200/Island |
| cg07169357 | chr2 | MFSD6/Body/- |
| cg02771240 | chr3 | -/-/S_Shore |
| cg26747536 | chr1 | -/-/- |
| cg16764274 | chr2 | SGPP2/Body/S_Shore |
| cg12564012 | chr7 | SDK1/Body/- |
| cg17924854 | chr7 | CRHR2,CRHR2,CRHR2,CRHR2,CRHR2/Body,Body,Body,Body,Body/- |
| cg06247406 | chr6 | GRIK2,GRIK2,GRIK2/TSS200,TSS200,TSS200/Island |
| cg12445693 | chr5 | LMNB1,LMNB1/TSS1500,TSS1500/N_Shore |
| cg12879183 | chr7 | -/-/- |
| cg25417988 | chr2 | MGAT4A,MGAT4A/TSS1500,Body/- |
| cg06602847 | chr9 | DBC1,DBC1/5'UTR,1stExon/Island |
| cg25106913 | chr6 | -/-/S_Shore |
| cg03230469 | chr5 | GDNF/5'UTR/Island |
| cg23314866 | chr19 | NAPA/Body/N_Shore |
| cg24346905 | chr1 | NTNG1,NTNG1,NTNG1/Body,Body,Body/Island |
| cg22603971 | chr15 | RASGRP1,RASGRP1,RASGRP1/Body,Body,Body/- |
| cg22353329 | chr17 | CBX4/TSS1500/Island |
| cg05337137 | chr4 | -/-/N_Shore |
| cg06624915 | chr14 | -/-/- |
| cg21849875 | chr5 | ADAMTS2/Body/N_Shore |
| cg24243287 | chr1 | ILDR2/Body/- |
| cg10626750 | chr16 | -/-/- |
| cg07544187 | chr19 | CILP2/Body/Island |
| cg14229264 | chr3 | -/-/- |
| cg04792813 | chr14 | CCDC85C/1stExon/Island |
| cg24029114 | chr6 | -/-/S_Shore |
| cg25950625 | chr5 | PLCXD3/Body/Island |
| cg10569244 | chr11 | CAPN5/5'UTR/- |
| cg12971683 | chr1 | ARID1A,ARID1A/Body,Body/- |
| cg21116900 | chr12 | SLC17A8,SLC17A8/TSS200,TSS200/- |
| cg07576142 | chr13 | GPC6/1stExon/Island |
| cg17331032 | chr9 | FBP1,FBP1/Body,Body/- |
| cg04528819 | chr7 | KLF14/1stExon/Island |
| cg14134497 | chr18 | DTNA,DTNA,DTNA/5'UTR,5'UTR,5'UTR/Island |
| cg26741530 | chr10 | C10orf32,C10orf32/TSS200,TSS200/N_Shore |
| cg21839260 | chr1 | -/-/- |
| cg27462685 | chr13 | LINC00347,LINC00347/Body,Body/- |
| cg25988759 | chr2 | -/-/- |
| cg21641634 | chr12 | NINJ2,NINJ2/Body,5'UTR/- |
| cg04347354 | chr3 | FAM19A4,FAM19A4/TSS1500,TSS1500/Island |
| cg03523785 | chr14 | FOXG1/TSS1500/Island |
| cg04063230 | chr7 | DNAJB6/Body/- |
| cg08102508 | chr3 | IQSEC1/TSS1500/Island |
| cg05009601 | chr7 | FEZF1,FEZF1,FEZF1,FEZF1/1stExon,1stExon,5'UTR,5'UTR/Island |
| cg03975694 | chr19 | ZNF540,ZNF540/5'UTR,1stExon/S_Shelf |
| cg18969232 | chr2 | -/-/Island |
| cg03095758 | chr1 | -/-/- |
| cg26161329 | chr17 | PPM1E/TSS1500/Island |
| cg04150927 | chr12 | -/-/- |
| cg05463027 | chr6 | KIF13A,KIF13A,KIF13A,KIF13A/TSS1500,TSS1500,TSS1500,TSS1500/S_Shore |
| cg13526756 | chr2 | -/-/S_Shore |
| cg03240067 | chr16 | -/-/- |
| cg07194694 | chr11 | -/-/- |
| cg17534046 | chr19 | -/-/- |
| cg11545034 | chr18 | -/-/- |
| cg16556679 | chr17 | SNORD7/TSS1500/- |
| cg09829319 | chr6 | GCM2/TSS200/N_Shore |
| cg20381975 | chr13 | -/-/Island |
| cg15149655 | chr2 | VWA3B,VWA3B/1stExon,5'UTR/Island |
| cg10831324 | chr15 | -/-/S_Shore |
| cg05675373 | chr1 | KCNC4,KCNC4,KCNC4/1stExon,1stExon,1stExon/Island |
| cg17992056 | chr1 | ICMT/TSS1500/S_Shore |
| cg05615459 | chr16 | -/-/- |
| cg22804528 | chr3 | -/-/- |
| cg10660256 | chr5 | BHMT,BHMT/5'UTR,1stExon/- |
| cg06488443 | chr2 | TBR1/Body/Island |
| cg09208331 | chr21 | -/-/Island |
| cg01976224 | chr3 | FAM19A4,FAM19A4/5'UTR,5'UTR/- |
| cg10521706 | chr2 | OSR1/5'UTR/Island |
| cg02793828 | chr16 | -/-/N_Shore |
| cg26692749 | chr1 | RCAN3/3'UTR/Island |
| cg25840576 | chr1 | TMEM200B/Body/Island |
| cg16336655 | chr3 | TMF1/TSS200/Island |
| cg03731131 | chr19 | KCNN4/Body/Island |
| cg24322623 | chr11 | MYOD1/TSS1500/N_Shore |
| cg23322172 | chr10 | TMEM180/Body/- |
| cg06641153 | chr18 | ST8SIA3/Body/S_Shore |
| cg09686672 | chr12 | LRP1/Body/- |
| cg06207035 | chr3 | LSAMP/Body/- |
| cg02574198 | chr14 | -/-/- |
| cg03685475 | chr22 | -/-/N_Shelf |
| cg01072786 | chr2 | GLB1L/Body/Island |
| cg09966085 | chr18 | SETBP1/Body/- |
| cg02954212 | chr16 | LOC283856,GNAO1,GNAO1/TSS1500,Body,Body/Island |
| cg00915126 | chr5 | BTNL9,BTNL9/Body,Body/- |
| cg16863013 | chr15 | AGBL1/Body/- |
| cg12232274 | chr6 | LRP11/TSS1500/S_Shore |
| cg00555393 | chr10 | -/-/- |
| cg27414593 | chr15 | SNORD116-9,SNORD116-3/TSS1500,TSS1500/- |
| cg05480055 | chr12 | SLC2A13/TSS1500/S_Shore |
| cg03598338 | chr16 | BCAR1,BCAR1,BCAR1,BCAR1,BCAR1/TSS200,TSS200,TSS200,Body,Body/Island |
| cg01191064 | chr5 | LOX/Body/N_Shelf |
| cg10575026 | chr11 | LOC101929295/Body/S_Shore |
| cg02452586 | chr1 | KIAA1804/Body/Island |
| cg16611727 | chr2 | OTOF,OTOF/TSS200,TSS200/N_Shelf |
| cg01743077 | chr11 | P2RY6,P2RY6,P2RY6,P2RY6,P2RY6,P2RY6,P2RY6,P2RY6/TSS1500,TSS200,1stExon,1stExon,1stExon,5'UTR,5'UTR,5'UTR/Island |
| cg26873338 | chr12 | CPSF6/Body/S_Shelf |
| cg11260097 | chr7 | -/-/Island |
| cg21215576 | chr8 | FAM83A,FAM83A/Body,Body/N_Shore |
| cg23600496 | chr2 | CCDC141/Body/- |
| cg27162435 | chr17 | KCNAB3/TSS1500/Island |
| cg23289226 | chr2 | MIR7853,NCKAP5,NCKAP5/Body,Body,Body/- |
| cg17413943 | chr11 | BDNF,BDNF,BDNF,BDNF,BDNF/Body,5'UTR,5'UTR,5'UTR,5'UTR/N_Shore |
| cg19558029 | chr18 | KATNAL2/TSS200/N_Shore |
| cg06268694 | chr22 | CELSR1/1stExon/Island |
| cg27625507 | chr11 | ETS1/5'UTR/- |
| cg08568868 | chr9 | USP20,USP20,USP20/5'UTR,5'UTR,5'UTR/- |
| cg00004608 | chr6 | -/-/N_Shore |
| cg24600422 | chr7 | TNPO3,TNPO3,TNPO3/Body,Body,Body/- |
| cg10037316 | chr14 | -/-/- |
| cg17587782 | chr10 | C10orf120/TSS200/- |
| cg17621913 | chr7 | -/-/- |
| cg19500220 | chr20 | DEFB115/TSS1500/- |
| cg12870987 | chr18 | -/-/N_Shore |
| cg08688023 | chr10 | PHYHIPL,CCHE1,PHYHIPL/TSS1500,TSS1500,Body/Island |
| cg26049390 | chr8 | -/-/- |
| cg19519393 | chr3 | GSK3B,GSK3B/Body,Body/N_Shore |
| cg17002328 | chr14 | CCDC88C/Body/N_Shelf |
| cg02754713 | chr15 | -/-/- |
| cg14272951 | chr16 | -/-/- |
| cg06290633 | chr10 | TACC2,TACC2,TACC2,TACC2/Body,Body,Body,Body/- |
| cg26516006 | chr10 | FGFR2,FGFR2,FGFR2,FGFR2,FGFR2,FGFR2,FGFR2,FGFR2,FGFR2,FGFR2,FGFR2/Body,Body,Body,Body,Body,Body,Body,Body,Body,Body,Body/- |
| cg01943200 | chr11 | CARD16,CARD16/TSS200,TSS200/- |
| cg15447912 | chr12 | GALNT9/Body/N_Shore |
| cg26007201 | chr14 | JKAMP,JKAMP,JKAMP,JKAMP,L3HYPDH,JKAMP,JKAMP,JKAMP,JKAMP/5'UTR,5'UTR,1stExon,5'UTR,TSS1500,ExonBnd,Body,Body,Body/S_Shore |
| cg10670616 | chr8 | TCF24,TCF24/5'UTR,1stExon/Island |
| cg21475536 | chr8 | GML/5'UTR/Island |
| cg20435485 | chr22 | FAM116B/Body/- |
| cg22798977 | chr1 | PDPN,PDPN/TSS1500,TSS1500/Island |
| cg12678274 | chr8 | -/-/- |
| cg05369911 | chr19 | -/-/- |
| cg02796545 | chr13 | KL/TSS200/Island |
| cg17705128 | chr19 | FUT6,FUT6,FUT6,FUT6/1stExon,1stExon,5'UTR,5'UTR/- |
| cg19600586 | chr20 | LINC00494/Body/- |
| cg22431130 | chr11 | -/-/- |
| cg01281539 | chr2 | -/-/- |
| cg11383809 | chr14 | SERPINA2,SERPINA2/Body,Body/- |
| cg02681442 | chr14 | FOXG1/TSS1500/N_Shore |
| cg14422984 | chr20 | -/-/- |
| cg10056132 | chr10 | C10orf53,C10orf53,C10orf53,C10orf53/5'UTR,5'UTR,1stExon,1stExon/Island |
| cg01862828 | chr8 | -/-/- |
| cg03399905 | chr15 | ANKRD34C/5'UTR/Island |
| cg23967169 | chr3 | FAM19A4,FAM19A4,FAM19A4,FAM19A4/1stExon,5'UTR,5'UTR,1stExon/Island |
| cg22993585 | chr5 | -/-/- |
| cg04898572 | chr22 | RBX1/Body/- |
| cg11101542 | chr2 | HAGLR,HOXD1,HAGLR,HAGLR,HAGLR,HAGLR,HAGLR/TSS1500,1stExon,TSS1500,TSS1500,TSS1500,TSS1500,TSS200/Island |
| cg08370178 | chr5 | -/-/N_Shelf |
| cg17033723 | chr5 | -/-/- |
| cg10877458 | chr6 | BRP44L/TSS1500/S_Shore |
| cg04431629 | chr1 | DMRTA2/TSS1500/Island |
| cg02064275 | chr17 | -/-/- |
| cg16997203 | chr11 | TP53I11,TP53I11/TSS200,TSS200/Island |
| cg07145843 | chr5 | PCSK1/TSS200/Island |
| cg03882167 | chr2 | FAM123C,FAM123C,FAM123C,FAM123C/5'UTR,5'UTR,5'UTR,5'UTR/Island |
| cg22694275 | chr15 | ANKRD34C/5'UTR/S_Shore |
| cg10996675 | chr20 | TPD52L2,TPD52L2,TPD52L2,TPD52L2,TPD52L2,TPD52L2,TPD52L2,TPD52L2,TPD52L2,TPD52L2,TPD52L2/TSS1500,TSS1500,TSS1500,TSS1500,TSS1500,TSS1500,TSS1500,TSS1500,TSS1500,TSS1500,TSS1500/S_Shore |
| cg00683788 | chr20 | PDYN/TSS200/- |
| cg20782778 | chr11 | YAP1,YAP1/Body,Body/Island |
| cg23361092 | chr13 | -/-/Island |
| cg01573554 | chr1 | NFYC,NFYC,NFYC,NFYC,NFYC,NFYC/TSS200,5'UTR,5'UTR,5'UTR,5'UTR,5'UTR/- |
| cg02857557 | chr10 | INPP5F,INPP5F/Body,TSS1500/S_Shore |
| cg23622047 | chr2 | RAPGEF4,RAPGEF4/Body,Body/- |
| cg23828720 | chr12 | HOXC9/TSS1500/Island |
| cg11257728 | chr22 | PARVG,PARVG,PARVG,PARVG,PARVG,PARVG,PARVG/5'UTR,Body,5'UTR,Body,Body,5'UTR,1stExon/- |
| cg03189652 | chr2 | PTH2R/TSS200/N_Shore |
| cg11248845 | chr19 | STRN4,STRN4,STRN4,STRN4/ExonBnd,ExonBnd,Body,Body/- |
| cg01552919 | chr4 | GAK/Body/S_Shore |
| cg16201354 | chr12 | HECTD4/Body/- |
| cg24662139 | chr22 | LINC01399/Body/- |
| cg20704342 | chr2 | C2orf3/TSS200/S_Shore |
| cg21046148 | chr5 | -/-/Island |
| cg13357249 | chr1 | PIP5K1A,PIP5K1A,PIP5K1A,PIP5K1A/TSS1500,TSS1500,TSS1500,TSS1500/N_Shore |
| cg00791406 | chr12 | GRIN2B/5'UTR/- |
| cg14454907 | chr2 | -/-/N_Shelf |
| cg21377521 | chr12 | TMTC1/Body/- |
| cg23163333 | chr2 | SPHKAP,SPHKAP/TSS200,TSS200/Island |
| cg02601039 | chr10 | LINC01516/Body/- |
| cg18131045 | chr5 | LOC643201/Body/- |
| cg24601522 | chr4 | -/-/- |
| cg14945834 | chr1 | SLC2A1,SLC2A1-AS1/TSS200,Body/S_Shore |
| cg18267374 | chr8 | NEFM,NEFM,NEFM/TSS1500,5'UTR,1stExon/Island |
| cg14315332 | chr3 | PCNP/Body/S_Shelf |
| cg08181572 | chr5 | OSMR,OSMR/5'UTR,5'UTR/- |
| cg24158052 | chr10 | -/-/- |
| cg07306469 | chr1 | -/-/- |
| cg15824707 | chr15 | DUOX2/Body/Island |
| cg05376185 | chr1 | MOBKL2C/TSS1500/S_Shore |
| cg07132386 | chr2 | ZDBF2,ZDBF2/TSS1500,TSS200/N_Shore |
| cg25088279 | chr8 | -/-/N_Shelf |
| cg01593013 | chr3 | PDIA5,PDIA5/Body,Body/- |
| cg00590036 | chr6 | TMEM181/TSS200/Island |
| cg03757784 | chr16 | HS3ST2/1stExon/Island |
| cg06034711 | chr8 | -/-/- |
| cg14504743 | chr14 | -/-/- |
| cg04094193 | chr4 | YTHDC1,YTHDC1/TSS1500,TSS1500/S_Shore |
| cg10659886 | chr19 | ZSCAN18,ZSCAN18/Body,1stExon/Island |
| cg07955995 | chr7 | KLF14/TSS1500/Island |
| cg08781559 | chr1 | ZYG11A,ZYG11A/5'UTR,Body/Island |
| cg22464292 | chr5 | PCDHGB5,PCDHGB5,PCDHGA8,PCDHGA1,PCDHGA2,PCDHGA3,PCDHGB1,PCDHGA4,PCDHGB2,PCDHGA5,PCDHGB3,PCDHGA6,PCDHGA7,PCDHGB4/TSS1500,TSS1500,Body,Body,Body,Body,Body,Body,Body,Body,Body,Body,Body,Body/Island |
| cg05937737 | chr3 | SLC7A14/5'UTR/Island |
| cg12306262 | chr10 | -/-/- |
| cg02866343 | chr16 | PPL/Body/- |
| cg13029847 | chr17 | SEZ6,SEZ6/TSS200,TSS200/S_Shore |
| cg24423515 | chr4 | ANKRD56/TSS200/Island |
| cg26799219 | chr8 | -/-/- |
| cg27632817 | chr17 | LINC00673/TSS200/Island |
| cg00924143 | chr12 | -/-/Island |
| cg00030816 | chr1 | RBM8A/TSS200/N_Shore |
| cg01908954 | chr15 | SCG3,SCG3,SCG3,SCG3/5'UTR,1stExon,5'UTR,1stExon/Island |
| cg18902090 | chr5 | PCDHA7,PCDHA12,PCDHA6,PCDHAC1,PCDHA10,PCDHA4,PCDHA11,PCDHA8,PCDHA6,PCDHA1,PCDHA2,PCDHA1,PCDHA9,PCDHA13,PCDHA5,PCDHA3,PCDHAC1,PCDHA10/Body,Body,Body,TSS200,Body,Body,Body,Body,Body,Body,Body,Body,Body,Body,Body,Body,TSS200,Body/Island |
| cg00833350 | chr10 | -/-/- |
| cg26913248 | chr2 | TWIST2/TSS1500/Island |
| cg22379378 | chr3 | -/-/- |
| cg15287138 | chr13 | -/-/- |
| cg23455677 | chr19 | ABCA7/Body/- |
| cg08380164 | chr13 | -/-/S_Shelf |
| cg14932454 | chr9 | -/-/- |
| cg10888390 | chr1 | UTP11L/TSS1500/Island |
| cg12833465 | chr14 | SFTA3/Body/Island |
| cg09177106 | chr11 | -/-/Island |
| cg02812207 | chr4 | PRSS12/1stExon/Island |
| cg23955764 | chr2 | -/-/- |
| cg19862427 | chr12 | MYL2/TSS200/- |
| cg10641011 | chr16 | -/-/- |
| cg13259925 | chr4 | LRAT/Body/Island |
| cg12781700 | chr17 | C17orf104/TSS200/Island |
| cg06265050 | chr3 | KCNAB1,KCNAB1,KCNAB1,KCNAB1,KCNAB1/Body,Body,Body,Body,Body/- |
| cg00349776 | chr1 | RXRG,RXRG,RXRG,RXRG/5'UTR,5'UTR,1stExon,1stExon/- |
| cg21781979 | chr13 | CLYBL/3'UTR/Island |
| cg26264372 | chr1 | LINC01141/Body/- |
| cg22070406 | chr18 | ST8SIA3/TSS200/Island |
| cg06218726 | chr14 | MIR203/TSS200/Island |
| cg11917125 | chr2 | -/-/- |
| cg18218371 | chr8 | RIMS2,RIMS2/5'UTR,1stExon/Island |
| cg12730047 | chr3 | -/-/- |
| cg06090569 | chr4 | UBE2K,UBE2K,UBE2K/Body,Body,Body/N_Shore |
| cg23950461 | chr3 | CCK/TSS1500/S_Shore |
| cg09165955 | chr5 | -/-/- |
| cg25236230 | chr15 | GPR176,GPR176/5'UTR,1stExon/Island |
| cg14506657 | chr9 | -/-/Island |
| cg04834436 | chr16 | IRX6/Body/Island |
| cg25934954 | chr11 | MAP4K2/Body/N_Shelf |
| cg18042724 | chr11 | SLC6A5/Body/S_Shelf |
| cg12613383 | chr1 | PLD5/TSS1500/Island |
| cg07476582 | chr2 | -/-/- |
| cg09662003 | chr6 | CFB,CFB/ExonBnd,Body/- |
| cg00631327 | chr8 | CSMD1/Body/- |
| cg20471798 | chr5 | SLC6A3/Body/- |
| cg15759112 | chr12 | LOC100507377/TSS1500/- |
| cg25580825 | chr19 | COL5A3/Body/N_Shelf |
| cg17165507 | chr19 | UNC13A,UNC13A/ExonBnd,Body/- |
| cg23821943 | chr4 | SORCS2/Body/- |
| cg00220737 | chr4 | KCTD8/Body/N_Shore |
| cg26923490 | chr19 | KCNA7/1stExon/Island |
| cg05531918 | chr19 | ZNF233,ZNF233/TSS200,TSS200/Island |
| cg23413924 | chr15 | DUOX1,DUOX1/Body,Body/Island |
| cg03539653 | chr12 | -/-/- |
| cg04965050 | chr22 | SCUBE1/TSS1500/Island |
| cg16952751 | chr2 | CFLAR,CFLAR,CFLAR/5'UTR,TSS200,1stExon/N_Shore |
| cg22484737 | chr7 | -/-/- |
| cg13902210 | chr1 | KCNC4,KCNC4,KCNC4/1stExon,1stExon,1stExon/Island |
| cg04784672 | chr14 | LRFN5,LRFN5/5'UTR,1stExon/S_Shore |
| cg12285737 | chr19 | -/-/Island |
| cg23937076 | chr20 | PTPRT,PTPRT,PTPRT,PTPRT/ExonBnd,ExonBnd,Body,Body/- |
| cg09571950 | chr3 | -/-/Island |
| cg24586039 | chr8 | RBPMS,RBPMS,RBPMS,RBPMS/Body,Body,Body,Body/- |
| cg26133164 | chr7 | -/-/- |
| cg13645221 | chr6 | PHACTR1,PHACTR1/Body,Body/- |
| cg13246235 | chr6 | PHACTR1/Body/Island |
| cg08222153 | chr1 | CROCC/TSS1500/- |
| cg07883149 | chr1 | RGS4,RGS4,RGS4,RGS4/TSS1500,TSS1500,5'UTR,1stExon/- |
| cg05546878 | chr2 | PRKAG3/TSS200/- |
| cg08319079 | chr2 | -/-/S_Shore |
| cg03430846 | chr8 | NRG1/TSS200/Island |
| cg13436155 | chr13 | -/-/S_Shore |
| cg11727282 | chr9 | ELAVL2/TSS1500/S_Shelf |
| cg00745389 | chr7 | -/-/Island |
| cg04747693 | chr16 | -/-/S_Shore |
| cg20399575 | chr7 | -/-/- |
| cg25927044 | chr10 | -/-/N_Shore |
| cg07006564 | chr15 | C2CD4A/Body/Island |
| cg05255275 | chr14 | C14orf39/5'UTR/Island |
| cg09727277 | chr7 | -/-/Island |
| cg25926549 | chr4 | MIR95,ABLIM2,ABLIM2,ABLIM2,ABLIM2,ABLIM2,ABLIM2,ABLIM2/TSS1500,Body,Body,Body,Body,Body,Body,Body/- |
| cg05571970 | chr6 | -/-/Island |
| cg06545198 | chr11 | -/-/- |
| cg13316116 | chr1 | -/-/- |
| cg06166341 | chr4 | CTBP1,CTBP1/Body,Body/N_Shore |
| cg22029941 | chr11 | DLG2,DLG2,DLG2/Body,Body,Body/- |
| cg25211403 | chr21 | -/-/Island |
| cg15873149 | chr3 | -/-/Island |
| cg20742415 | chr1 | METTL11B/TSS1500/- |
| cg25136068 | chr20 | ZHX3/5'UTR/- |
| cg12284854 | chr1 | -/-/N_Shelf |
| cg13920491 | chr3 | ATP13A4/Body/- |
| cg18517534 | chr10 | CCDC7/Body/- |
| cg13918518 | chr17 | C17orf82/TSS1500/Island |
| cg00617867 | chr5 | PCDHA6,PCDHA2,PCDHA1,PCDHA9,PCDHA7,PCDHA1,PCDHA6,PCDHA5,PCDHA10,PCDHA3,PCDHA4,PCDHA10,PCDHA8/Body,Body,Body,Body,Body,Body,Body,Body,Body,Body,Body,Body,Body/Island |
| cg11892980 | chr16 | -/-/N_Shelf |
| cg03398055 | chr17 | MSI2,MSI2/Body,Body/- |
| cg06746968 | chr8 | -/-/- |
| cg09583957 | chr20 | GNAS,GNAS,GNAS,GNAS,GNAS/5'UTR,5'UTR,1stExon,1stExon,3'UTR/Island |
| cg03453449 | chr12 | USP44,USP44/1stExon,5'UTR/S_Shelf |
| cg21982455 | chr5 | -/-/S_Shore |
| cg13449967 | chr11 | NA |
| cg17916490 | chr2 | NTSR2/1stExon/Island |
| cg16298598 | chr19 | CHST8,CHST8,CHST8/Body,Body,Body/- |
| cg18274065 | chr18 | CYB5A,CYB5A,CYB5A/TSS1500,TSS1500,TSS1500/S_Shore |
| cg04865692 | chr19 | KCNC3/1stExon/Island |
| cg18798248 | chr6 | HIST1H1B/TSS200/Island |
| cg03061778 | chr10 | -/-/- |
| cg03032214 | chr18 | GALR1/TSS200/Island |
| cg03925395 | chr8 | TRAPPC9,TRAPPC9/Body,Body/- |
| cg02094774 | chr6 | -/-/- |
| cg03060590 | chr7 | -/-/- |
| cg02625638 | chr8 | FAM83A,FAM83A/Body,Body/N_Shore |
| cg09392068 | chr17 | -/-/S_Shore |
| cg00158333 | chr17 | IGF2BP1,IGF2BP1/Body,Body/Island |
| cg25820491 | chr1 | SDCCAG8/Body/Island |
| cg19474047 | chr19 | ANGPTL6/5'UTR/S_Shore |
| cg13476469 | chr7 | -/-/- |
| cg01039763 | chr9 | -/-/Island |
| cg25432913 | chr14 | BRF1,BRF1,BRF1,BRF1,BRF1,PACS2/TSS1500,TSS1500,TSS1500,5'UTR,5'UTR,5'UTR/S_Shore |
| cg26649752 | chr12 | SRRM4/TSS1500/N_Shore |
| cg18954541 | chr20 | SLC13A3,SLC13A3/1stExon,5'UTR/Island |
| cg22743483 | chr12 | RIMBP2/Body/S_Shore |
| cg09745331 | chr4 | ENPP6/Body/- |
| cg26471191 | chr2 | MIR7853,NCKAP5,NCKAP5/Body,Body,Body/- |
| cg09498007 | chr2 | CREG2/TSS1500/S_Shore |
| cg03969651 | chr17 | TBX4/Body/- |
| cg02238388 | chr1 | SYPL2/TSS200/Island |
| cg14839404 | chr20 | BMP7/TSS200/S_Shore |
| cg04630292 | chr6 | PRRT1/3'UTR/Island |
| cg16911220 | chr12 | KRT86/1stExon/Island |
| cg07799295 | chr20 | EYA2,EYA2/5'UTR,5'UTR/- |
| cg18446045 | chr7 | VGF/TSS1500/Island |
| cg15593298 | chr3 | PAQR9/1stExon/Island |
| cg07963349 | chr17 | GALR2/Body/Island |
| cg04991447 | chr5 | SEMA6A,SEMA6A,SEMA6A,SEMA6A/1stExon,1stExon,5'UTR,5'UTR/Island |
| cg08470639 | chr9 | NTRK2,NTRK2,NTRK2,NTRK2,NTRK2/5'UTR,5'UTR,5'UTR,5'UTR,5'UTR/Island |
| cg24158129 | chr16 | -/-/- |
| cg26118906 | chr14 | -/-/Island |
| cg08381504 | chr3 | -/-/Island |
| cg04940570 | chr11 | TEAD1/5'UTR/Island |
| cg07212449 | chr3 | VGLL4,VGLL4,VGLL4/Body,Body,Body/- |
| cg04514834 | chr5 | -/-/- |
| cg14911690 | chr19 | PBX4/1stExon/Island |
| cg08801007 | chr1 | NID1/Body/- |
| cg08888412 | chr16 | DHX38,TXNL4B,TXNL4B,DHX38,TXNL4B,TXNL4B/1stExon,5'UTR,5'UTR,5'UTR,TSS200,1stExon/S_Shore |
| cg03834286 | chr5 | -/-/N_Shore |
| cg25922163 | chr19 | GGN/TSS1500/Island |
| cg00247855 | chr5 | FSTL4/Body/- |
| cg12639146 | chr8 | RGS20,RGS20,RGS20,RGS20,RGS20,RGS20,RGS20/Body,Body,Body,Body,Body,Body,Body/- |
| cg05372434 | chr10 | ZNF25/TSS1500/S_Shore |
| cg14707092 | chr19 | TMEM38A/Body/S_Shore |
| cg10906284 | chr12 | AVPR1A/1stExon/Island |
| cg04760426 | chr1 | SLC45A1/Body/Island |
| cg17069396 | chr20 | EBF4/Body/Island |
| cg06726490 | chr2 | NEURL3/Body/N_Shore |
| cg16430256 | chr10 | -/-/- |
| cg03672274 | chr4 | MSX1/TSS1500/N_Shore |
| cg04563870 | chr11 | TNNT3,TNNT3,TNNT3,TNNT3,TNNT3/5'UTR,5'UTR,5'UTR,5'UTR,5'UTR/- |
| cg06572160 | chr19 | KCNC3/1stExon/Island |
| cg02888131 | chr15 | ALDH1A2,ALDH1A2/TSS200,TSS200/Island |
| cg19296405 | chr19 | KIAA1683,KIAA1683,KIAA1683/Body,Body,Body/Island |
| cg12389336 | chr2 | KDM3A,KDM3A/Body,Body/- |
| cg13358573 | chr4 | CORIN,CORIN,CORIN/Body,Body,Body/- |
| cg17422915 | chr11 | KIRREL3,KIRREL3/Body,Body/- |
| cg24378439 | chr18 | -/-/S_Shore |
| cg09444979 | chr15 | SLC30A4,C15orf21/TSS1500,Body/Island |
| cg15970686 | chr10 | TACC2,TACC2,TACC2,TACC2/Body,Body,Body,Body/- |
| cg16061668 | chr11 | CTTN,CTTN/Body,Body/N_Shore |
| cg08620606 | chr19 | SELV/TSS200/N_Shore |
| cg07924081 | chr19 | PPP1R13L,PPP1R13L/Body,Body/Island |
| cg11699476 | chr8 | CRH/TSS200/S_Shore |
| cg04414675 | chr7 | PTPRN2,PTPRN2,PTPRN2/Body,Body,Body/Island |
| cg13526492 | chr19 | CYP2B6/Body/- |
| cg13486406 | chr7 | -/-/Island |
| cg01580888 | chr19 | RHPN2/TSS1500/Island |
| cg04354077 | chr20 | PROKR2/TSS200/N_Shore |
| cg00511674 | chr16 | -/-/Island |
| cg12034641 | chr14 | FBXO34,FBXO34/5'UTR,5'UTR/- |
| cg01214817 | chr2 | -/-/- |
| cg19996355 | chr19 | PBX4/1stExon/Island |
| cg03730958 | chr6 | FOXQ1/TSS200/Island |
| cg11806672 | chr13 | POU4F1/Body/Island |
| cg03101422 | chr2 | 0 |
