# Supplementary material for: An aging and p53 related marker: HOXA5 promoter methylation negatively correlates with mRNA and protein expression in old age
Source: Aging (Albany NY). 2021 Feb 5;13(4):4831–49. doi: 10.18632/aging.202621 (PMC7950283; doi:10.18632/aging.202621)
Supplement: Supplementary Table 2 [file aging-13-202621-s002.pdf]

SUPPLEMENTARY TABLE

Supplementary Table 2. Region-specific primers applied for single CpG analysis.

Gene	Number of CpGs	Forward primer	Reverse primer
HOXA5	14	5`-AGCTTCCTTCCTGTTCTGGG-3`	5`TCAGAGACACTAGCACAGGAGCC-3`
DUSP3	4	5`-TCCATCACCATGGGTGGAAGGCA-3`	5`-CTGTCAGTCAAAGAAAGGGA-3`
RYR1	25	5`-TCCCTGTAGGAGCCTGGACA-3`	5`-GGAACCCCAGCCAGTGAGAA-3`
